# Haplotype-resolved genome assembly provides insights into evolutionary history of the tea plant *Camellia sinensis*

**DOI:** 10.1038/s41588-021-00895-y

**Published:** 2021-07-15

**Authors:** Xingtan Zhang, Shuai Chen, Longqing Shi, Daping Gong, Shengcheng Zhang, Qian Zhao, Dongliang Zhan, Liette Vasseur, Yibin Wang, Jiaxin Yu, Zhenyang Liao, Xindan Xu, Rui Qi, Wenling Wang, Yunran Ma, Pengjie Wang, Naixing Ye, Dongna Ma, Yan Shi, Haifeng Wang, Xiaokai Ma, Xiangrui Kong, Jing Lin, Liufeng Wei, Yaying Ma, Ruoyu Li, Guiping Hu, Haifang He, Lin Zhang, Ray Ming, Gang Wang, Haibao Tang, Minsheng You

**Affiliations:** 1 State Key Laboratory of Ecological Pest Control for Fujian and Taiwan Crops, Institute of Applied Ecology, College of Plant Protection, Fujian Agriculture and Forestry University, Fuzhou, China; 2 Shenzhen Branch, Guangdong Laboratory for Lingnan Modern Agriculture, Genome Analysis Laboratory of the Ministry of Agriculture, Agricultural Genomics Institute at Shenzhen, Chinese Academy of Agricultural Sciences, Shenzhen, China; 3 Institute of Rice, Fujian Academy of Agricultural Sciences, Fuzhou, China; 4 Center for Genomics and Biotechnology, Fujian Provincial Key Laboratory of Haixia Applied Plant Systems Biology, Key Laboratory of Genetics, Fujian Agriculture and Forestry University, Fuzhou, China; 5 Ministerial and Provincial Joint Innovation Centre for Safety Production of Cross-Strait Crops, Joint International Research Laboratory of Ecological Pest Control (Ministry of Education), Fujian Agriculture and Forestry University, Fuzhou, China; 6 Tobacco Research Institute, Chinese Academy of Agricultural Sciences, Qingdao, China; 7 Hangzhou Kaitai Biotech Co. Ltd, Hangzhou, China; 8 Department of Biological Sciences, Brock University, St. Catharines, Ontario Canada; 9 Key Laboratory of Tea Science, College of Horticulture, Fujian Agriculture and Forestry University, Fuzhou, China; 10 Tea Research Institute, Fujian Academy of Agricultural Sciences, Fuzhou, China; 11 Jiangxi Sericulture and Tea Research Institute, Nanchang, China; 12 Key Laboratory of Cultivation and Protection for Non-Wood Forest Trees, Ministry of Education, Central South University of Forestry and Technology, Changsha, China; 13 Department of Plant Biology, University of Illinois at Urbana-Champaign, Urbana, IL USA; 14 CAS Key Laboratory of Tropical Forest Ecology, Xishuangbanna Tropical Botanical Garden, Chinese Academy of Sciences, Mengla, China

**Keywords:** Population genetics, Genomics, Plant genetics

## Abstract

Tea is an important global beverage crop and is largely clonally propagated. Despite previous studies on the species, its genetic and evolutionary history deserves further research. Here, we present a haplotype-resolved assembly of an Oolong tea cultivar, Tieguanyin. Analysis of allele-specific expression suggests a potential mechanism in response to mutation load during long-term clonal propagation. Population genomic analysis using 190 *Camellia* accessions uncovered independent evolutionary histories and parallel domestication in two widely cultivated varieties, var. *sinensis* and var. *assamica*. It also revealed extensive intra- and interspecific introgressions contributing to genetic diversity in modern cultivars. Strong signatures of selection were associated with biosynthetic and metabolic pathways that contribute to flavor characteristics as well as genes likely involved in the Green Revolution in the tea industry. Our results offer genetic and molecular insights into the evolutionary history of *Camellia sinensis* and provide genomic resources to further facilitate gene editing to enhance desirable traits in tea crops.

## Abstract (web summary)

Haplotype-resolved genome assembly of an Oolong tea cultivar Tieguanyin and population genomic analyses of 190 *Camellia* accessions provide insights into the evolutionary history of the tea plant *Camellia sinensis*.

## Main

Many agronomically important crops are clonally propagated, including potato, cassava and tea. Such clonal propagation can be effective to maintain valuable genotypes that may segregate or be lost through sexual recombination^1^. However, this method has some disadvantages, including greater vulnerability to crop loss through shared disease susceptibility. Clonal crops can be prone to accumulating deleterious mutations, leading to high mutation load in plants that reproduce asexually due to ‘Muller’s ratchet’^2^. High levels of deleterious mutations in individuals can ultimately reduce relative fitness, associated with reduction of agronomic performance^1^. Diplontic selection can purge deleterious mutations and involves selecting specific cells bearing favorable alleles from a mixture of other cell lineages^1,3^. However, evolutionary consequences of mutation load in clonally propagated crops remain unclear.

Tea, produced from *C. sinensis*, is a widely consumed beverage that contains multiple polyphenolic compounds considered beneficial to human health^4^. Although the origin of tea drinking is unclear^5^, archeological evidence from the Mausoleum of Han Yangling indicates that tea drinking was popular by the 2nd century BCE during the Western Han dynasty^6^. With more than two billion cups consumed every day, tea is an extremely important crop economically and globally, yielding an annual global harvest of ~5 million tons, worth about US $5.7 billion (ref. ^7^). Tea is classified into two varieties, *C. sinensis* var. *sinensis* (CSS) and var. *assamica* (CSA) with a number of distinct features, such as leaf size^8^. Both varieties are flavorful, carry health-promoting bioactive compounds and have been domesticated for commercial tea production.

Recent studies have provided reference genomes for the two varieties^9–11^; however, the mosaic assemblies likely missed allelic variations underlying important selected traits. One of the studies of the tea genome generated a phased assembly based on construction of a genetic map. This strategy required a large effort to perform resequencing and variant calling of 135 sperm cells^12^, hindering application to other crops. Population structure and genetic diversity in tea plants have been extensively discussed recently^9–11^, which substantially contributed to the study of tea genomics. Nevertheless, the complex evolutionary history and uncertain phylogeny, especially the reticulate evolutionary pattern with wild close relatives, remain to be examined.

Tea plants exhibit allogamy and self-incompatibility^13^. This leads to a high level of heterozygosity in the genome, providing a model to investigate allelic variations that may play important roles during evolution. Hybridization among variable tea cultivars is known to produce offspring with desirable traits superior to both parents, indicating the importance of heterosis in tea breeding^14^. Abundant germplasm resources and the well-documented pedigree of cultivars make this species an attractive model system for studying the mechanism underlying heterosis. Here we show a chromosome-scale genome for the Chinese Oolong tea variety Tieguanyin (TGY; Chinese for ‘Iron Goddess of Mercy’), with two haplotypes fully represented. We also resequenced several leading tea accessions and close relatives to explore genetic diversity among geographically distinct tea populations. Our results provide insight into the mechanism of heterosis and the evolutionary history of the tea plant and uncover important signatures of selection.

## Results

### Genome assembly and annotation

**Extended Data Fig. 1:**
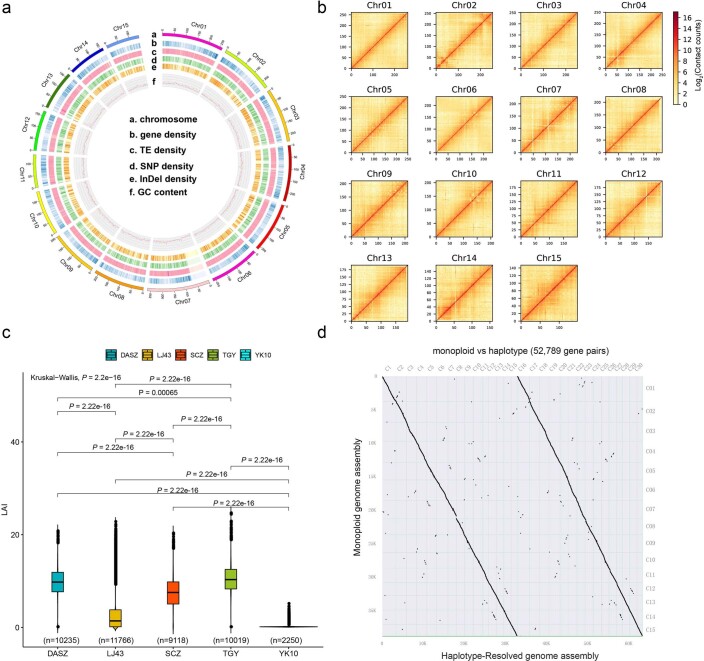
Genome feature and assessment of assemblies along the sequenced Oolong tea chromosomes (‘TGY’). **a**, The circles (from outermost to innermost) represent monoploid genome in Mb, gene density, TE density, SNP density, indel density and GC content. **b**, Genome-wide analysis of chromatin interactions at 1 Mb resolution in the TGY genome. **c**, Assessment of five tea genome assemblies using LTR Assembly Index (LAI), which are DASZ, LJ43, SCZ, TGY and YK10. The x-axis lists names of these cultivars and y-axis represents LAI values. In each box plot, the bold line in centre indicates median value and bounds of box are the first (25%) and third (75%) quantiles. The minima and maxima values are present in the lower and upper whiskers, respectively. *P* values were calculated using the two-sided Kruskal-Wallis test without multiple comparison. **d**, Syntenic analysis of TGY monoploid genome assembly and TGY haplotype-resolved genome assembly.

**Extended Data Fig. 2:**
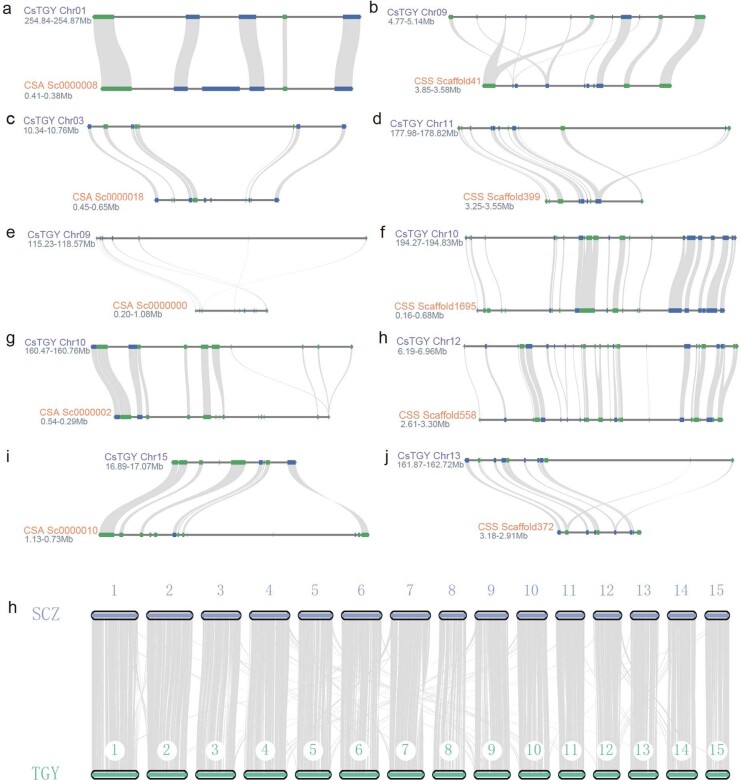
Synteny analysis between TGY monoploid genome assembly with CSA-YK10 and CSS-SCZ assemblies. The top 20 longest scaffolds from CSA-YK10 genome and CSS-SCZ genome were extracted for the snyteny analysis and only five of them were randomly selected for visualization **a-j**. Synteny analysis was also shown between TGY and SCZ genomes at chromosome level **k**.

**Extended Data Fig. 3:**
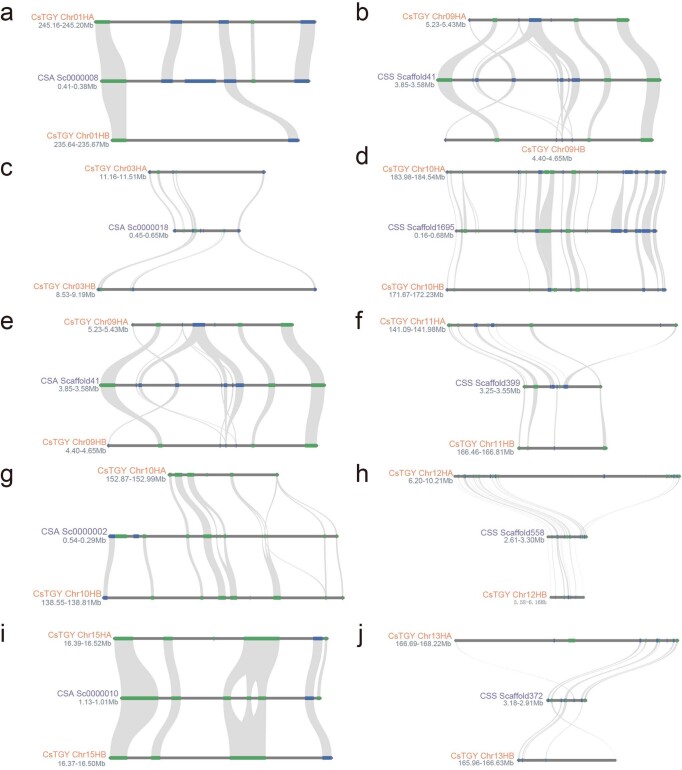
Synteny analysis between TGY haplotype-resolved genome assembly with CSA and CSS assemblies. The top 20 longest scaffolds from CSA genome and CSS genome were extracted for the snyteny analysis and only five of them were randomly selected for visualization **a-j**.

The genome size of TGY was estimated to be ~3.15 Gb with a heterozygosity of 2.31%. Our initial contig-level assembly using 359 Gb (114×) of PacBio long reads was 5.41 Gb (Table 1), indicating high heterozygosity levels across the genome. Heterozygous sequences were identified using a new program (Khaper^15^) based on *k*-mer counting (Supplementary Note 1 and Supplementary Fig. 1). Comparison between our algorithm and existing programs revealed that Khaper is highly efficient and fast and handles heterozygous diploid species with large genome sizes (Supplementary Table 1). In total, 2.35 Gb of sequences were filtered from the initial contig assembly, resulting in a 3.06-Gb monoploid assembly with a contig *N*_50_ of 1.94 Mb and 93.7% benchmarking universal single-copy ortholog (BUSCO) completeness for the monoploid genome (Table 1). The resulting contigs were corrected using chromatin contact patterns in 3D-DNA^16^ and linked into 15 pseudo-chromosomes that anchored 3.03 Gb (98.96%) of the monoploid genome (Extended Data Fig. 1a and Supplementary Tables 2 and 3). This monoploid genome represented a mosaic assembly of the two haplotypes, which selected the longest allelic contigs from the Canu^17^ initial assembly. Assessment of genome assembly using a series of approaches validated a high-quality reference assembly of the TGY genome (Supplementary Note 2, Supplementary Tables 4 and 5 and Extended Data Figs. 1–3).

**Table 1:** Summary of genome assembly and annotation of *C. sinensis* TGY

Sequencing	*C. sinensis* cultivar TGY	
PacBio Sequel II sequencing		
Raw data (Gb)	359	
Sequencing depth (×)	114	
Average reads length (bp)	1,608	
Reads *N*_50_ (bp)	24,830	
Hi-C sequencing		
Clean data (Gb)	313	
Sequencing depth (×)	99.4	
**Monoploid genome assembly and annotation**		
Estimated genome size (Gb) per 1 *C*	3.15	
Assembly size (Gb)	3.06	
Percent of estimated genome size (%)	97.1	
Contig *N*_50_ (Mb)	1.94	
BUSCO completeness of assembly (%)	93.7	
Total number of genes	42,825	
BUSCO completeness of annotation (%)	92.1	
**Haplotype-resolved chromosomal-level assembly and annotation**		
	**Haplotype A**	**Haplotype B**
Length of chromosomes (Gb)	3.06	2.92
BUSCO completeness of assembly (%)	84.8	83.2
BUSCO completeness of annotation (%)	85.0	82.4
Number of genes with annotated alleles^a^	32,596	24,723
Number of genes with two alleles^a^	14,691	
Number of genes with one allele^a^	27,937	
Total number of anchored genes	42,628	
Unanchored genes or alleles	197	

^a^Only one allele was retained if the two allelic genes had the exact same coding sequences.

We predicted 42,825 protein-coding genes, collectively showing 92.1% BUSCO completeness (Table 1 and Supplementary Table 6). We also identified 2.39 Gb of repetitive sequences, accounting for 78.2% of the monoploid genome (Supplementary Table 7). A total of 20,969 intact long terminal repeats (LTRs) were identified in the TGY genome (Supplementary Table 8). A very recent LTR retrotransposon burst event was detected in the genome, dating back to 0.3–0.5 million years ago (Ma), based on the divergence of the terminal sequences of the repeats (Extended Data Fig. 4).

### Haplotypic variations and allelic imbalance

**Fig. 1:**
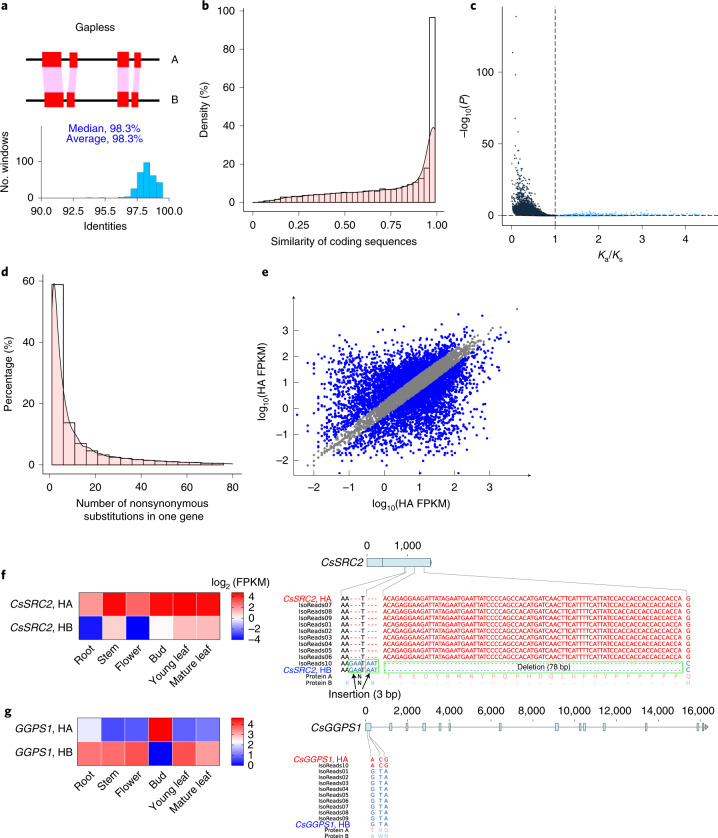
Genetic variations between haplotypes and allelic imbalance in *C. sinensis*. **a**, Whole-genome comparison of two haplotypes using 10-Mb non-overlapping windows with no gap extension allowed within alignment blocks. The distribution of identities is shown in the lower panel, with the *x* axis representing identities and the *y* axis indicating the number of windows supporting the corresponding identity. **b**, Pairwise comparison of coding sequences for alleles. **c**, Pairwise comparison of the *K*_a_/*K*_s_ distribution for allelic genes. Blue dots indicate genes with *K*_a_/*K*_s_ > 1, and black dots indicate genes with *K*_a_/*K*_s_ < 1. *P* values were calculated using a two-sided Fisher’s exact test. **d**, Numerical distribution of nonsynonymous substitutions between alleles. **e**, Identification of ASEGs in leaves. Coordinates are logarithmically scaled (log_10_). Blue dots indicate ASEGs, and gray dots represent genes that are not ASEGs. FPKM, fragments per kb exon per million fragments mapped. HA, haplotype A; HB, haplotype B. **f**, An example of an ASEG (*CsSRC2*) with a consistent expression pattern across tissues. Left, allelic differential expression of this gene in six tissues (stem, bud, root, flower, young leaves and mature leaves). Right, allelic variations between haplotype A and haplotype B, including one nonsynonymous mutation, two 3-bp insertions and one 78-bp insertion, which are supported by Iso-seq reads. The deduced amino acids resulting from these allelic variations are shown in the alignments and are indicated by protein A for haplotype A and protein B for haplotype B. **g**, An example of an ASEG (*CsGGPS*) with an inconsistent expression pattern. Left, differential allele expression of this gene in the six tissues above. Right, three nonsynonymous allelic variations supported by a number of Iso-seq reads.

The high level of heterozygosity in the TGY genome allowed us to phase two haplotypes using ALLHiC^18^. Collapsed contigs were identified and duplicated based on read depth (Supplementary Note 2), recovering 564 Mb of homozygous sequences. The augmented set of sequences was subjected to haplotype phasing along with Canu phased contigs, resulting in a fully haplotype-solved assembly with 30 pseudo-chromosomes and 5.98 Gb of sequences anchored (Table 1 and Supplementary Table 9). Syntenic analysis revealed highly consistent gene order in both haplotypes (Extended Data Fig. 1d). To investigate sequence divergence and evolutionary relationships, we stringently aligned genome sequences with no gaps or indels allowed within an alignment block, finding 98.3% sequence identity between the two haplotypes (Fig. 1a). We also detected 3.7 million SNPs, 118,700 insertions and 118,335 deletions (Supplementary Table 10). These variations spanned 101.7 Mb, representing 3.3% of the assembled monoploid genome. The two haplotypes contained similar levels of repetitive sequences (74.3% in haplotype A and 74.2% in haplotype B; Supplementary Table 11). Estimation of switch errors^19^ relying on phased SNPs (Methods) showed an error rate of 5.9% (8,473 of 144,868), likely resulting either from the contig assembly or ALLHiC phasing. We observed that the haplotype-resolved assembly contained substantially fewer switch errors than the monoploid assembly (23.6%, 94,273 of 399,821), indicating that our phasing approach is vastly superior to existing approaches that only create a chimeric monoploid genome.

Using these phased haplotypes, we separated 34.5% (14,691 of 42,628) of the annotated genes with two defined alleles (Table 1). Most allelic genes maintained high levels of coding sequence similarity (mean = 93%; Fig. 1b), and a vast majority of allelic genes underwent purifying selection, with an average *K*_a_/*K*_s_ ratio of 0.07 (Fig. 1c). We further identified large-effect allelic variations that may influence gene function, including one pair with start codon loss, one pair with stop codon loss, 297 pairs with premature stop codons and 719 pairs with frame shifts. In total, 86.9% of allelic gene pairs contained at least one nonsynonymous substitution (Fig. 1d). These differences indicate that our haplotype-phased TGY assembly uncovers structural and functional allelic differences.

**Extended Data Fig. 5:**
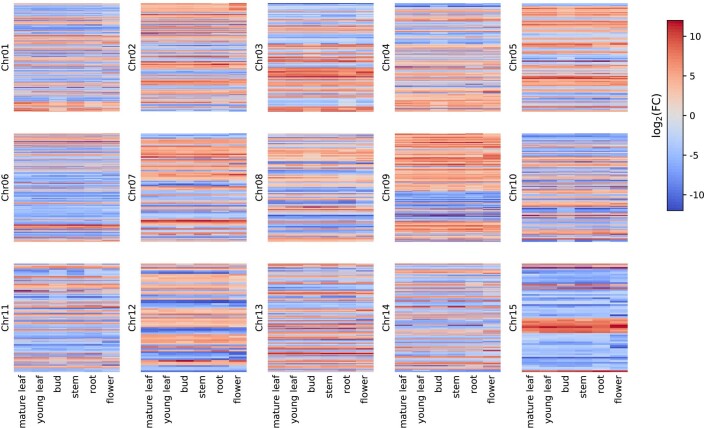
Genes with consistent allele-specific expression (ASE) pattern across six tissues of bud, root, stem, flower, young and mature leaves. The color bar represents log_2_(FC) values. FC indicates fold change of FPKM values between allele A and allele B. Red color suggests that expression in allele A is significantly higher than allele B and blue color means that expression in allele B is significantly higher than allele A.

We next investigated allelic imbalance, that is, allele-specific expression (ASE), without resequencing the parental genomes. We found that 30.1% of genes (4,423 of 14,691) showed significant ASE in tea leaves (*P* < 0.05 and false discovery rate <0.05; Fig. 1e), indicating consistent and inconsistent allelic expression patterns. A comparison of 14,691 allele-defined genes resulted in 1,528 genes with expression biased toward one allele (that is, consistent ASE genes (ASEGs)) across the six tissues (Extended Data Fig. 5 and Supplementary Table 12). These genes showed functional enrichment in multiple biological processes, including ribosome, endocytosis, basal transcription factor and spliceosome Kyoto Encyclopedia of Genes and Genomes (KEGG) pathways (Supplementary Fig. 2), suggesting that a potential mechanism to overcome deleterious mutations occurred in important genes related to basic biological functions. For instance, the *CsSRC2* gene showed a consistent expression pattern across the six tested tissues. The ortholog in *Arabidopsis* *thaliana* encodes an activator of a calcium-dependent pathway that mediates reactive oxygen species production in response to cold stress^20^. We observed two 3-bp insertions and one 78-bp deletion in the second exon of haplotype B, introducing two additional amino acids (lysine and asparagine) while removing 26 amino acids from the deduced protein sequences (Fig. 1f). A nonsynonymous mutation was also detected in haplotype A, from G to C in haplotype B, modifying the amino acid from glutamine to histidine. These allelic variations were further supported by several Iso-seq reads.

**Extended Data Fig. 6:**
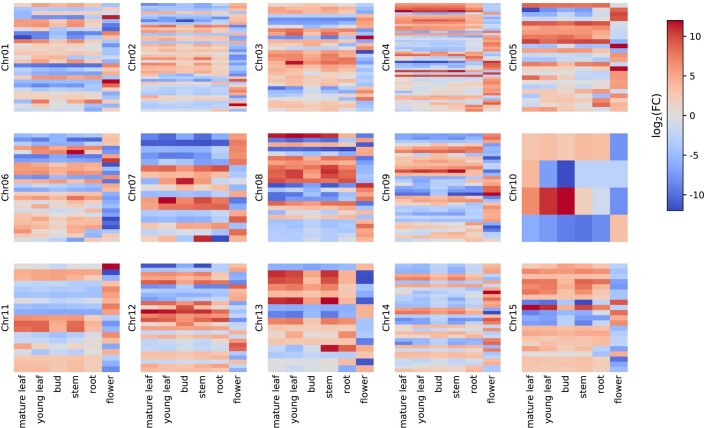
Genes with inconsistent allele-specific expression (ASE) pattern (direction shifting) across six tissues of bud, root, stem, flower, young and mature leaves. The color bar represents log_2_(FC) values. FC indicates fold change of FPKM values between allele A and allele B. Red color suggests that expression in allele A is significantly higher than allele B, and blue color means that expression in allele B is significantly higher than allele A.

In addition to consistent ASEGs, we found 386 inconsistent ASEGs that displayed switched high expression between alleles in different tissues (Extended Data Fig. 6). Several of these genes were associated with biosynthesis of volatile organic compounds, including flavone and flavonol, terpenoid backbone and falvonoid biosynthesis pathways (Supplementary Table 13). For example, the *CsGGPS* gene, encoding geranylgeranyl diphosphate synthase, plays an important role in terpenoid backbone biosynthesis. A comparison of the two alleles showed 99.0% similarity; meanwhile, three amino acids were modified by three nonsynonymous mutations (Fig. 1g).

### Patterns of genetic variation and population structure

**Fig. 2:**
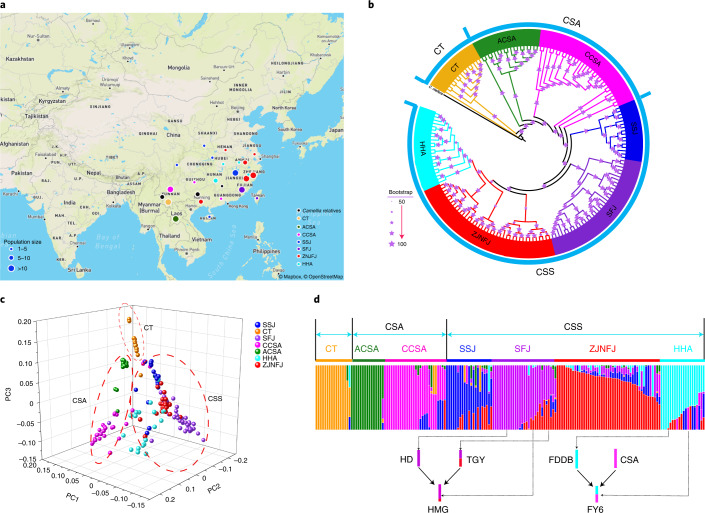
Phylogenetic relationships and population structure of resequenced individual tea plants. Accessions are represented in the same color code throughout this figure (black, wild close relatives; orange, CT or *C. taliensis*; green, ACSA; pink, CCSA; dark blue, SSJ; purple, SFJ; red, ZJNFJ; cyan, HHA). **a**, Geographic distribution of resequenced individual tea plants. Population sizes are indicated by circle sizes. Base map © OpenStreetMap (https://www.openstreetmap.org/copyright). **b**, Maximum-likelihood tree with bootstrap values supported. Larger sizes of asterisks indicate higher values of bootstraps, mostly close to 100%. **c**, PCA of resequenced individual tea plants. PC, principal component. **d**, Ancestry results from Admixture under the *k* = 7 model supported by an examination of cross-validation errors (Extended Data Fig. 7). Two documented modern breeding events are indicated below the Admixture plot (HD, Huangdan; HMG, Huangmeigui; FDDB, Fudingdanbai; FY6, Fuyun 6).

We resequenced 129 *Camellia* accessions collected from 15 provinces across four major tea-growing regions: southwest of China, south of the Yangtze River, south of China and north of the Yangtze River (Fig. 2a). Along with 61 recently published resequenced tea samples, a total of 190 *Camellia* accessions were used in our analysis, containing 113 CSS, 48 CSA, one *C. sinensis* var. *pubilimba*, 15 *Camellia taliensis*, 12 closely related species and one *Camellia oleifera* as the outgroup (Supplementary Table 14). A total of 7.26 Tb of sequences with an average depth of 12.75× per accession were generated (Supplementary Table 14) and mapped onto the monoploid assembly, identifying 9,407,149 SNPs and 829,388 small indels (<10 bp) (Table 2).

**Table 2:** Summary of genetic variation in tea populations

Category	Core set	*C. taliensis*	ACSA	CCSA	SSJ	SFJ	ZJNFJ	HHA
Sequence variants								
SNPs	9,407,149	1,634,833	5,520,700	8,058,883	8,089,989	8,734,917	8,462,225	8,190,015
Indels (<10 bp)	829,388	144,805	474,306	706,576	703,295	763,808	739,147	716,033
Variants with effects on genes								
SNPs that introduce stop codons	10,925	2,259	6,424	9,341	9,131	9,943	9,518	9,215
SNPs that disrupt stop codons	1,033	221	633	909	907	965	949	924
SNPs that induce alternative splicing	3,788	802	2,258	3,252	3,198	3,489	3,332	3,202
Indels located in genic regions	207,235	39,635	119,726	177,496	179,152	192,450	186,738	180,503
Frameshift indels	12,570	2,834	7,450	10,678	10,598	11,513	11,075	10,672
Genes affected by large-effect variants	9,136	2,577	6,221	8,221	8,134	8,610	8,401	8,184
Nonsynonymous variants	290,638	65,736	177,108	250,001	250,952	269,739	260,287	251,814
Synonymous variants	194,509	44,744	120,383	168,743	170,440	181,399	175,497	169,732

Ratios of nonsynonymous to synonymous SNPs in tea accessions were almost exactly the same, ranging from 1.47 to 1.49 (Supplementary Table 15). We analyzed large-effect SNPs that might impact gene function, including gain or loss of a stop codon or changes potentially affecting alternative splice sites (Table 2). In total, 9,136 protein-coding genes contained large-effect SNPs, and 207,235 indels were identified in genic regions, with 12,570 (6.07%) introducing frame shifts (Table 2). Functional analysis highlighted the binding function in gene ontology (GO) terms and plant–pathogen interaction in KEGG pathways (Supplementary Figs. 3 and 4), linking large-effect mutations to evolutionary adaptation.

**Extended Data Fig. 7:**
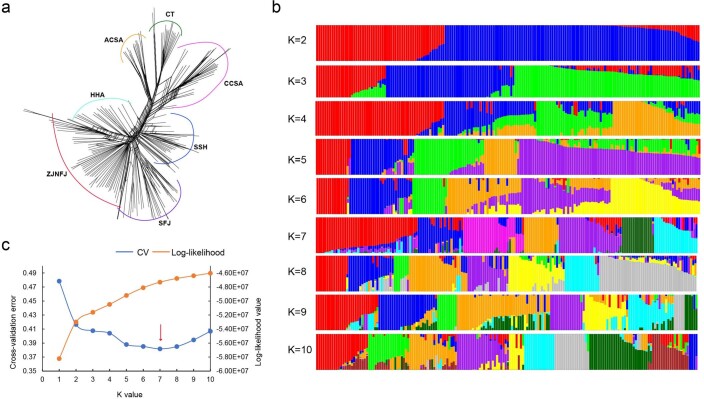
Analysis of popualtion structure, envolutinoary and LD decay. Evolutionary relationship of different tea populations based on network analysis using splitsTree. **b**, Population structure inferred by Admixture analysis of 176 tea accessions (*K* = 2 to 10). **c**, Cross-validation error shows that K = 7 is the optimal population clustering group.

Phylogenetic analysis using 496,448 SNPs located in single-copy genes separated a subset of *Camellia* samples including 15 of *C. taliensis* and 161 of *C. sinensis* into three major types: *C. taliensis*, CSA and CSS*,* with *C. taliensis* being the most closely related to the outgroup (Fig. 2a–c). We observed two subgroups in the CSA type: ancestral CSA (ACSA) and cultivated CSA (CCSA). The ACSA subgroup represented samples collected from regions far from human territory (that is, wild forest) and clustered at the base of the cultivated tea accessions. The CSS group was partitioned into four subgroups, which are named after their dominant geographic locations: SSJ (Sichuan, Shaanxi and Jiangxi), SFJ (south Fujian), ZJNFJ (Zhejiang and north Fujian) and HHA (Hubei, Hunan and Anhui). Hierarchical structures were observed within some subgroups, such as SFJ, presumably due to frequent genetic exchanges among different subgroups according to our Admixture results (Fig. 2d). Results from network analysis using SplitsTree^21^ were in agreement with the maximum-likelihood tree; however, they showed a more complex network of phylogenetic relationships (Extended Data Fig. 7a). The first three axes of the principal-component analysis (PCA) further confirmed this population structure but showed more divergence between ACSA and CCSA subgroups (Fig. 2c).

**Extended Data Fig. 8:**
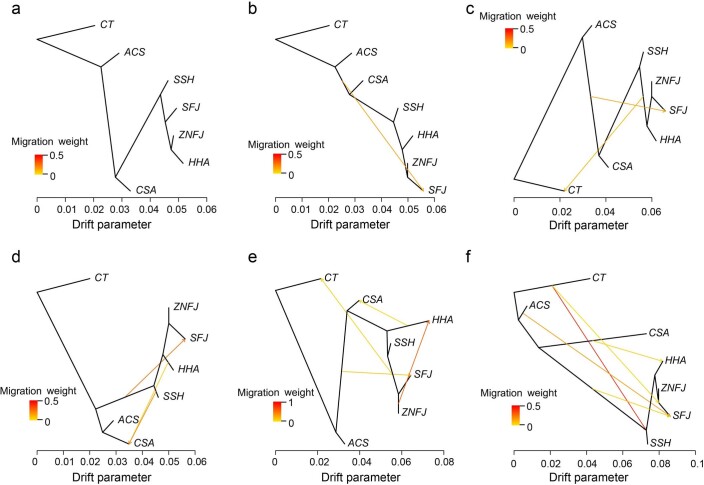
TreeMix analysis of allelic drift among different groups of tea populations. Best-fitting genealogy for the tea populations calculated from the variance-covariance matrix of genome-wide allele frequencies. The lines with arrows indicates possible migration events. Color scale represent the weight of migration. and the scale bar indicates 10 times the average SE of the relatedness among populations based on the variance–covariance matrix of allele frequencies.

Genetic clustering analysis revealed an optimal value of *k* = 7 subpopulations with the lowest cross-validation errors supported, consistent with the population structure derived by maximum-likelihood tree and PCA (Fig. 2 and Extended Data Fig. 7b,c). TreeMix analysis identified significant gene flow among these tea populations (Extended Data Fig. 8), indicating frequent intraspecific introgression, likely due to historical germplasm exchanges. Our population structure analysis reasonably showed consistency of genetic and geographic distribution of these tea germplasms. The Admixture^22^ plot detected the occurrence of a series of historical hybridization as well as documented modern breeding events. For instance, TGY and Huangdan are ancestors of several elite tea cultivars^14^, such as Huangmeigui. We observed that Huangmeigui (red and purple) was mixed, with a substantial contribution of genetic material originated from or similar to Huangdan (purple) and TGY (red and purple). In addition, the Admixture analysis is consistent with the documented breeding event that Fuyun 6 is a typical descendant of Fudingdabai^11^, showing a mixture of Fudingdabai (cyan) and one unknown CSA accession (pink) in Fuyun 6 (pink and cyan; Fig. 2d).

**Extended Data Fig. 9:**
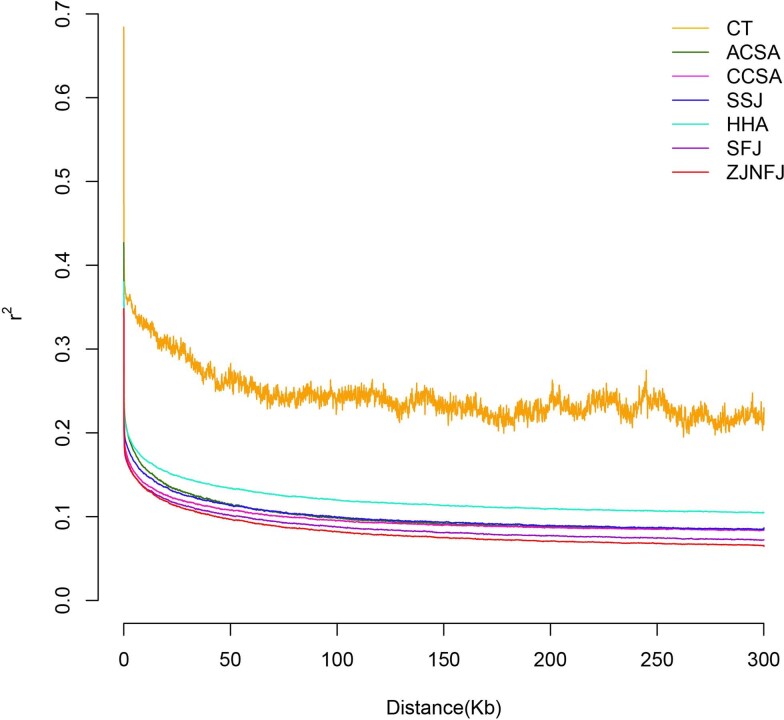
Decay of linkage disequilibrium (LD) in each of the geographic groups. CT represents *C. taliensis*; ACSA is ancestral *C. sinensis* var. *assamica*; CCSA means cultivated *C. sinensis* var. *assamica*; SSJ indicates samples from Sichuan, Shaanxi and Jiangxi; SFJ means South Fujian; ZJNFJ indicates Zhejiang and North Fujian; HHA include samples from Hubei, Hunan and Anhui.

We observed a slightly higher nucleotide diversity (*π*) within the CCSA subgroup (6.44 × 10^−4^) than that within the four CCSS populations (average *π* = 6.22 × 10^−4^; Supplementary Table 15) and a similar level of linkage disequilibrium decay among the six subgroups compared to a rapid decay over physical distance in the wild *C. taliensis* group (Extended Data Fig. 9 and Supplementary Table 16). We further calculated population fixation statistics (*F*_ST_) to investigate population divergence, which showed that the population divergence among four CSS subgroups (average = 3.67 × 10^−2^) was much smaller than that between the two CSA subgroups (8.77 × 10^−2^; Supplementary Table 17). We observed similar *F*_ST_ values when comparing the *C. taliensis* group to each of the six tea subgroups. On the other hand, the four CCS subgroups showed smaller population divergence from CCSA than that from ACSA.

### Evolutionary history and genetic introgression

**Fig. 3:**
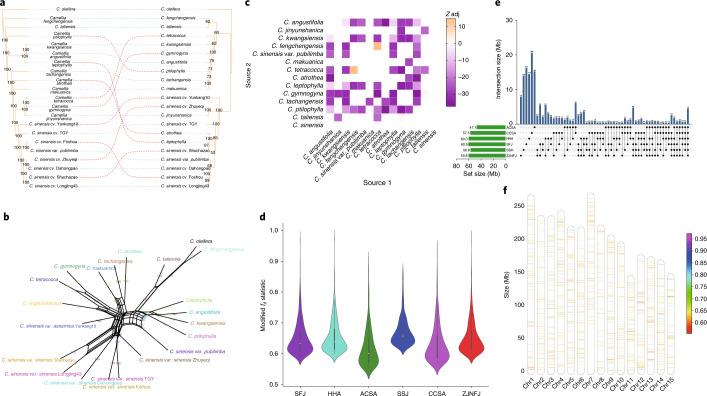
Genome-wide patterns of genetic introgression to modern tea cultivars from their close relatives. **a**, Cytonuclear conflicts between nuclear and chloroplast phylogenetic trees among 14 resequenced *Camellia* section *Thea* species with *C. oleifera* included as the outgroup. **b**, SplitsTree network for *Camellia* accessions from section *Thea*. **c**, Detection of introgression events between *C. sinensis* and close relatives using the *f*_3_ test. *Z* scores were adjusted based on a Benjamini–Hochberg false discovery-rate correction method, and significant introgression is indicated with purple if adjusted (adj) *Z* score < −1.96. **d**, Distribution of 95th percentile *f*_d_ outliers using modified *f*_d_ statistics (*y* axis) in six groups of cultivated tea populations (*x* axis). The white dot in the center of each violin plot represents the median value, and the bounds of each box indicate first (25%) and third (75%) quartiles. Minima and maxima are present in the lower and upper bounds of the whiskers, respectively, and the width of whiskers are densities of modified *f*_d_ statistics. *P* values were calculated using two-sided Fisher’s exact test without multiple comparisons. **e**, Amount of unique and shared introgressed sequences (in Mb) among six groups of cultivated tea populations. **f**, Distribution of introgressed loci along chromosomes (chr) 1–15, with the colored bar indicating the maximum of modified *f*_d_ statistics in each 100-kb non-overlapping window.

To investigate tea evolutionary history, we collected 12 close relatives from *Camellia* section *Thea*, the same section as *C. sinensis*. Along with eight selected *C. sinensis* accessions (including six CSS, one CSA and one var. *pubilimba*) and one outgroup, 21 individual plants from 14 *Camellia* species were resequenced at the whole-genome level (Supplementary Table 14). Based on a set of 9,407,149 high-quality SNPs, we observed that the eight *C. sinensis* accessions were clustered in a single group (Fig. 3a). Phylogenetic network analysis using SplitsTree^19^ supported the phylogenetic relationship in section *Thea* but illustrated a complex pattern of reticulate evolution (Fig. 3b).

We observed discordance between 500 sampled individual gene trees and a species tree constructed using ASTRAL-III^23^ (Supplementary Fig. 5). Frequent cytonuclear conflicts between nuclear and chloroplast trees were also detected (Fig. 3a), supporting the reticulate evolution likely associated with hybridization. To determine the genetic introgression occurring between *C. sinensis* and its close relatives, we performed the *f*_3_ test for each triplet (a combination of P1, P2 and P3) within the species from section *Thea* with *C. sinensis* as P3. The *f*_3_ analysis showed significant adjusted negative *Z* scores (adjusted Z score < −1.96) in most tested triplets (Fig. 3c), indicating that extensive hybridization events, rather than incomplete lineage sorting, contributed to the complex evolutionary history of *C. sinensis*.

We further screened introgressed loci in cultivated tea by calculating the modified *f*_d_ value^24^ and identified 1,485 genomic regions, comprising 172.2 Mb of sequences and 5.6% of the monoploid genome. The six geographic groups of cultivated tea populations displayed similar levels of introgressed sequences (Fig. 3d; ACS, 47.1 Mb; CSA, 57.5 Mb; HHA, 60.0 Mb; SFJ, 60.5 Mb; SSJ, 56.8 Mb; ZJNFJ, 59.8 Mb); however, only 2.6% (4.5 of 172.2 Mb) were shared. Each group had a large proportion (an average of 26.1%) of unique introgression loci, indicating independent introgression events during the parallel domestication of each population (Fig. 3e). In total, 98 genes were located in the 4.5-Mb regions, and these were significantly enriched in specific biological processes (*Q* < 0.05), including transporting ATPase activity and metalloexopeptidase activity (Supplementary Fig. 6).

**Extended Data Fig. 10:**
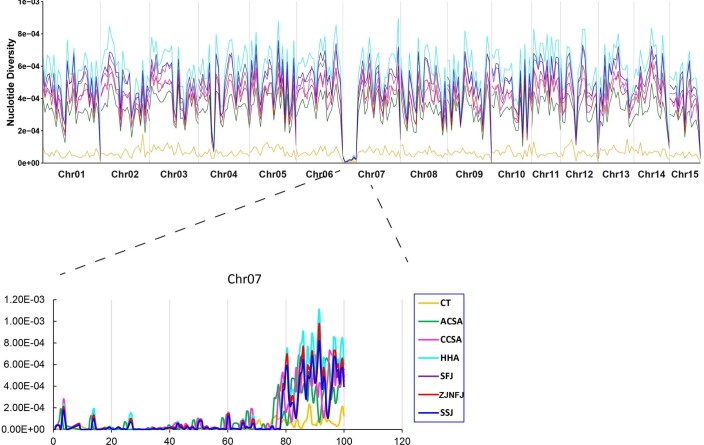
**Profiling of nucleotide diversity of** ***C. sinensis*** **populations showing an extremely low nucleotide diversity in 0–20** Mb and 40–50 Mb of chromosome 07.

Introgressed loci were not evenly distributed across different chromosomal regions (Fig. 3f). For instance, a large 50-Mb region in chromosome 7 displayed no introgression region. We observed extremely low *π* values in *C. sinensis* populations (Extended Data Fig. 10) and low heterozygosity in its close relatives (Supplementary Fig. 7) in 0–20 Mb and 40–50 Mb of this region, indicating a population bottleneck event or genetic hitchhiking due to natural selection in section *Thea*.

**Fig. 4:**
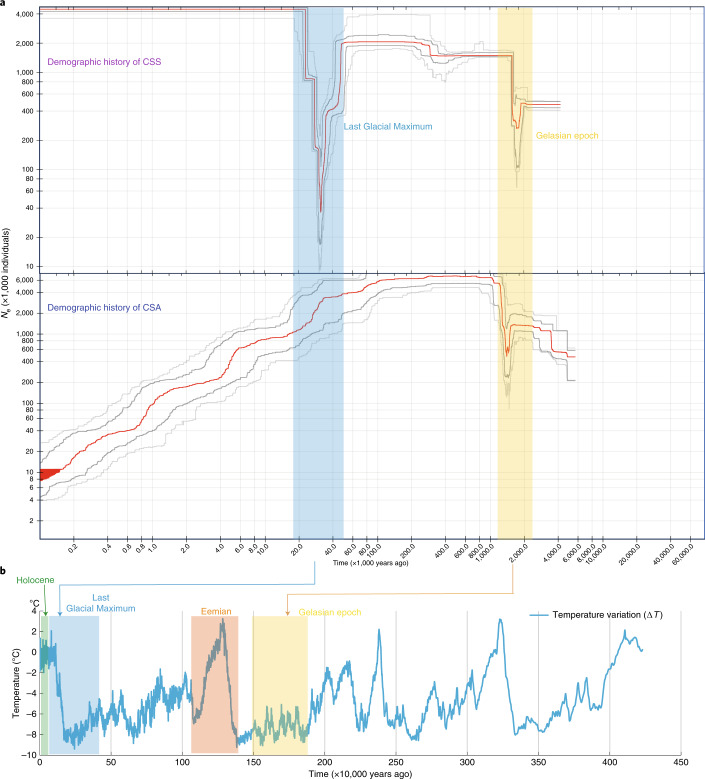
Demographic history of CSS and CSA. **a**, Historical effective population size *N*_e_ for CSS (top) and CSA (bottom). Stairway plot showing that *C. sinensis* underwent two bottlenecks during the known periods of major climate upheaval: the Gelasian epoch and the Last Glacial Maximum. The first one is shared by both CSS and CSA, and the second one is unique to CSS. **b**, Specific presentation of cultivated populations with ice core data for the past 4000,000 years (ref. ^25^).

Analysis of demographic history by estimating historical effective population size (*N*_e_) showed that *C. sinensis* underwent two demographic bottlenecks, both coinciding with known periods of environmental change (Fig. 4). The first bottleneck event, observed for both CSS and CSA, maps to a dramatic temperature decline in the Gelasian epoch^25^ (2.59–1.81 Ma). However, the second *N*_e_ drop was restricted to CSS and occurred during the extremely low temperatures^25^ of the Last Glacial Maximum (26,500–19,000 years ago), followed by a rapid demographic expansion (Fig. 4). This analysis indicated a different evolutionary history after divergence between CSA and CSS.

### Evidence of parallel domestication in CSA and CSS

**Fig. 5:**
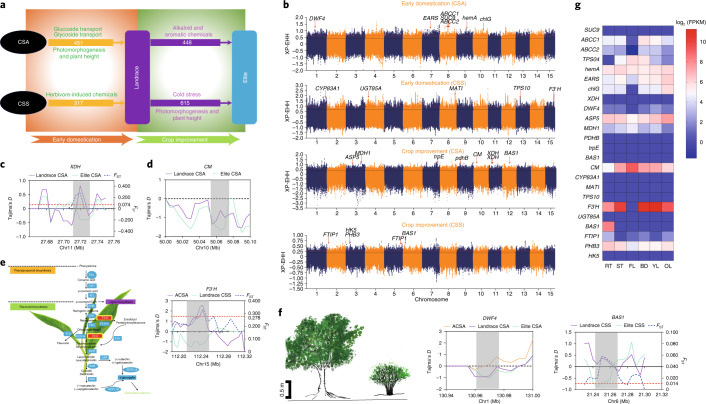
Signatures of artificial selection and evidence of parallel domestication in CSA and CSS. **a**, A proposed road map of parallel domestication in CSA and CSS. Early domestication involved genes related to glucoside and glycoside transport and photomorphogenesis and plant height in CSA and herbivore-induced chemicals in CSS. The improvement mainly focused on genes related to alkaloid and aromatic chemicals in CSA and genes related to cold stress and photomorphogenesis and plant height in CSS. The number of artificially selected genes are labeled in each domestication process. **b**, Genome-wide distribution of selective-sweep signals identified based on cross-population extended haplotype homozygosity (XP-EHH). Genes with important functions that are linked to sweeps are highlighted in Manhattan plots. Genes that were involved in early domestication were identified based on a comparison between CSA and CSS landraces, and ACSA, while genes under improvement were selected based on a comparison between CSA and CSS elite, and CSA and CSS landraces. **c**, Signals of artificial selection in the *XDH* gene. Purple and green solid lines indicate Tajima’s *D* statistics in CSA landraces and CSA elite cultivars, respectively. The blue dashed line indicates the fixation index (*F*_ST_) between CSA landrace and elite populations, while the red dashed line is the threshold of the top 5% *F*_ST_. **d**, Signals of artificial selection in the *CM* (chorismate mutase) gene. Purple and green solid lines indicate Tajima’s *D* statistics in CSA landraces and CSA elite cultivars, respectively. **e**, Signals of artificial selection in the *F3*′*H* gene. Left, *F3*′*H* is one of the key genes in catechin biosynthetic pathways. Right, signals of artificial selection in this gene. **f**, Signals of artificial selection in *BAS1* and *DWF4* genes, related to plant height. The leftmost panel shows different morphological features in tea accessions. In contrast to ACSA, CCSA and CCSS are featured with decreased plant height, with most CCSA being small trees or semi-shrubs and CCSS being shrubs. Scale bar, 0.5 m. Middle and rightmost panels show signals of artificial selection in *BAS1* and *DWF4*. **g**, Expression of artificially selected genes in the six tissues examined, including root (RT), stem (ST), flower (FL), bud (BD), young leaf (YL) and old leaf (OL).

To investigate genes related to early domestication and improvement in tea plants, we classified the CCSA and CCSS tea accessions into landraces and elite cultivars. Elite cultivars possess several highly desirable traits and have been certificated by the National Crop Variety Approval Committee in China. The remaining accessions were considered as landraces, while the ACSA served as the wild population with limited artificial selection. Based on stringent thresholds (Methods), we identified that 451 and 317 protein-coding genes were artificially selected in the early domestication processes in CSA and CSS landraces, respectively. Meanwhile, comparisons between landraces and elite cultivars revealed 448 and 615 genes under crop improvement, respectively (Fig. 5a,b). Collectively, 874 and 920 genes were domesticated in CSA and CSS, respectively; however, only 95 were shared, strongly suggesting parallel domestication processes for CSA and CSS.

Functional analysis showed that these domesticated genes were associated with a series of important biological processes. In the early domestication of CSA, the selected genes were significantly enriched for GO terms including glucoside transport, glycoside transport and (+)-abscisic acid d-glucopyranosyl ester transmembrane transport (*Q* < 0.01; Supplementary Fig. 8). The improvement process in CSA focused mainly on genes related to metabolism and biosynthesis of alkaloid and aromatic chemicals, including caffeine and pyruvate metabolism and phenylalanine, tyrosine and tryptophan biosynthesis, based on KEGG analysis (*P* < 0.05; Supplementary Fig. 9). The *CsXDH* gene, encoding xanthine dehydrogenase–oxidase, involved in a caffeine-related pathway, showed significantly low Tajima’s *D* values in elite CSA accessions and a high *F*_ST_ score above the threshold (Fig. 5c). In addition, we observed an obvious difference in Tajima’s *D* values between CSA landraces and elite CSA in a *CM* (chorismate mutase) gene (Fig. 5d), leading to biosynthesis of aromatic amino acids in the shikimate pathway^26^.

The early domestication of CSS cultivars involved genes associated with plant defense against insects and herbivores (Supplementary Fig. 10). Meanwhile, these selected genes were also significantly enriched in biosynthesis of important secondary metabolites, including (*R*)-limonene, (*E*)-β-ocimene, pinene, myrcene and α-farnesene (*P* < 0.05 and *Q* < 0.05; Supplementary Figs. 11 and 12). This result suggested that herbivore-induced chemicals were likely targets during the early domestication of CSS landraces. The improvement process from landraces to elite cultivars mainly focused on genes significantly enriched in regulation of flower development and response to nitric oxide (NO; *P* < 0.05 and *Q* < 0.05; Supplementary Fig. 13). Compared to CSA, CSS showed enhanced tolerance to cold stress and was therefore able to adapt to a relatively wide range of areas. A previous study showed that NO increased cold tolerance in tea plants by accelerating the consumption of γ-aminobutyric acid^27^, suggesting that these domesticated genes related to the response to NO likely conferred tolerance to cold stress in CSS.

Two domestication processes selected genes with important biological functions. *F3*′*H*, involved in catechin biosynthesis, showed strong artificial selection, supported by a high *F*_ST_ score and a significantly low Tajima’s *D* statistic in CSS landraces compared to those of ACSA accessions (Fig. 5b,e). Two genes encoding cytochrome P450 (*CsCYP734A1* (*CsBAS1*) and *CsCYP90B1* (*CsDWF4*)), associated with photomorphogenesis, were also under artificial selection in the early domestication of CSA and the improvement process of CSS, respectively (Fig. 5f), likely contributing to reduced plant height in cultivated tea accessions. RNA-seq analysis further supported the potential functions of these selected genes in six different tissues (Fig. 5g).

## Discussion

TGY is a world-renowned Oolong tea cultivar, which was selected during the reign of Yongzheng Emperor in the Qing Dynasty (1,723–1,735 A.D.). A ~300-year clonal propagation has led to accumulation of substantial somatic mutations in the genome, allowing us to separate the two haplotypes using our newly developed algorithms (Khaper^15^ and ALLHiC^18^) and identify allelic imbalance. ASEGs were classified into two major patterns: consistent ASEGs and inconsistent ASEGs (that is, a direction-shifting pattern). Consistent ASEGs had an allele with biased expression across all the tested tissues of tea plants, supporting a dominance effect on heterosis. Genes with expression biased toward one parental allele in some samples but shifted to another allele in other samples (that is, inconsistent ASEGs) indicate an overdominance effect^28^. In contrast to hybrid rice^28^, we observed considerably more consistent ASEGs than inconsistent ASEGs (1,528 versus 386) in *C. sinensis*, suggesting that the dominance effect played a major role in the highly heterozygous tea genome. The large number of consistent ASEGs is likely caused by accumulation of somatic mutations due to the long period of clonal propagation in tea plants. Study of the mechanism of widespread ASE possibly due to epigenetic modifications^29–32^ is a further work that deserves much effort. Basing on our results, we propose that the dominance effect likely provides a potential mechanism to overcome mutation load in clonally propagated tea plants.

The two ancient bottlenecks in CSS, both coinciding with a dramatic temperature decline, should lead to a substantial reduction in population diversity and smaller *N*_e_ values compared to those of CSA, which only experienced one bottleneck. However, the reduced diversity in CSS was likely counterbalanced by extensive introgression over its evolutionary history. Phylogenetic analysis revealed a reticulate evolution due to extensive inter- and intraspecific introgression in section *Thea*. Pervasive introgression contributed to the high level of genetic diversity in CSS populations and possibly enhanced adaptation to diverse environments, leading to a rapid demographic expansion after the second bottleneck. A large number of modern tea accessions are clonally propagated, and the accumulation of somatic mutations also contributes to increased diversity in other crops, such as grapes^33^. A comparison between two TGY samples collected from Fujian and Anhui revealed a high level of genetic difference (0.71%), even in the same cultivar.

Our efforts to detect signatures of artificial selection provided evidence of parallel domestication in CSA and CSS. The two varieties possess distinct features, such as various aromatic chemicals, different plant heights and cold tolerance, which were likely targets of artificial selection over the domestication history. Our results uncovered that several protein-coding genes associated with these economically important traits underwent domestication. Key genes related to biosynthetic metabolism of alkaloid and aromatic chemicals, including caffeine and catechins, contributed to the feature of interest in tea plants. In contrast to ACSA, CCSA and CCSS have reduced plant height, with CSA being small trees or semi-shrubs and CSS being shrubs. The morphological modification (plant height) in CCSA and CCSS is likely associated with domestication, as two cytochrome P450 genes (*CsCYP734A1* (*CsBAS1*) and *CsCYP90B1* (*CsDWF4*)) associated with photomorphogenesis were under artificial selection in CSS and CSA cultivars, respectively. These two genes are involved in brassinosteroid biosynthesis. Loss of function in the *Arabidopsis dwf4* mutant results in dwarfism due to abnormal cell elongation^34^, while the double mutant in *BAS1* along with its functionally redundant paralog (*SOB7*) displays elongated hypocotyl and decreased sensitivity to light^35^. Similar to wheat *Rht* genes and the rice *sd1* gene^36^, the two genes *CsBAS1* and *CsDWF4* likely contributed to the Green Revolution in tea industry as they may have introduced dwarfing traits into this crop. In conclusion, this study provides important insights into genome evolution, allelic imbalance, population genetics and further directions for crop breeding of tea plants. Our newly developed genomic resources can advance molecular biology research and ultimately offer tools and knowledge for shortening the 20–25-year breeding cycle through gene-targeted improvement of the tea crop.

## Methods

### Sample collection and DNA sequencing

The TGY plant used for PacBio sequencing and de novo genome assembly was maintained by the Tea Research Institute, Fujian Academy of Agricultural Sciences. Leaves were collected from a single TGY individual, planted in the county of Anxi located in Fujian Province, China (119.576708 E, 27.215297 N). In addition, we constructed a comprehensive dataset by incorporating our resequenced data (129 samples) as well as recently published 61 non-redundant resequenced accessions^9^. A total of 190 *Camellia* accessions were used in the present study, containing 113 CSS, 48 CSA, one *C. sinensis* var. *pubilimba*, 15 *C. taliensis*, 12 closely related species and one *C. oleifera* as the outgroup. These tea accessions consisted of 51 elite cultivars, 92 landraces, 18 ancestral tea accessions and 12 wild closely related species from section *Thea*. Young leaves from each accession were flash frozen in liquid nitrogen and transferred to the DNA sequencing provider (Annoroad Gene Technology) in Beijing. Genomic DNA from each sample was isolated using the DNeasy Plant Mini kit (Qiagen) following the manufacturer’s instructions. For PacBio long-read sequencing, we first applied the BluePippin system for size selection. SMRTbell libraries (30–50 kb) were then constructed according to the protocol released from PacBio. A total of three single-molecule real-time cells were sequenced on a PacBio Sequel II platform, generating 359 Gb of subreads. DNA samples that were used for whole-genome resequencing were sequenced using the Illumina NovaSeq platform with a read length of 150 bp and an insert size of 300–500 bp. In addition, the 10x Genomics library was constructed using high-molecular-weight DNA (>50 kb) according to the manufacturer’s protocol (https://support.10xgenomics.com/de-novo-assembly/library-prepr/doc/user-guide-chromium-genome-reagent-kit-v1-chemistry). Reads of approximately 300 Gb were sequenced on the Illumina NovaSeq platform with the 150-bp paired-end sequencing model.

### Genome assembly and annotation

We assembled the TGY genome by incorporating Illumina short-read sequences and PacBio single-molecule real-time long-read sequences as well as sequences from high-throughput chromatin conformation capture (Hi-C) technologies. A total of 359 Gb (~114× coverage) of subreads generated from the PacBio Sequel II platform were subjected to self-correction, trimming and assembly. All three steps were accomplished using Canu^17^ (version 1.9) with optimized parameters designed for polyploid genomes to assemble heterozygous genome sequences as far as possible (batOptions, ‘-dg 3 -db 3 -dr 1 -ca 500 -cp 50’). To further correct systematic errors of PacBio sequencing, we generated ~183 Gb (58× coverage) of Illumina short reads from the same TGY individual. These short reads were mapped against the Canu initial genome assembly using BWA^37^ MEM with default parameters, and variants that were considered to result from sequencing errors were polished using Pilon^38^ with parameters ‘--mindepth 4 --threads 6 --tracks --changes --fix bases --verbose’.

We provided two levels of chromosome-scale assemblies, including a monoploid genome and a haplotype-resolved assembly. For the monoploid genome, we first used our newly developed program Khaper to select primary contigs and filter redundant sequences (Supplementary Note 1) from the initial Canu assembly. Results were then inspected based on BUSCO completeness and duplication score. Meanwhile, we constructed two high-quality Hi-C libraries using previously described methodology^39^. Chimeric DNA fragments that represented sequences from proximal regions were sequenced on the Illumina NovaSeq platform with the paired-end model. The resulting non-redundant contigs were subjected to ALLHiC scaffolding with a diploid model^18^ and then partitioned into 15 groups, representing 15 pseudo-chromosomes. The chromosome number and orientation were renamed according to the chromosome-scale assembly of CSS-SCZ published previously^9^ for comparison. For the haplotype-resolved genome assembly, we first detected misassembled contigs that displayed abnormal long-range contact patterns from paired-end read alignments against the Canu initial assembly using Juicer tools^40^ and the 3D-DNA pipeline^16^, and only the first round of Hi-C corrected contigs were retained for haplotype phasing. We further applied a read-depth strategy to identify and duplicate collapsed contigs in the Canu initial assembly (that is, phased contigs) (Supplementary Note 2). Along with the duplicated sequences, Canu phased contigs were subjected to haplotype phasing using the ALLHiC^18^ polyploid scaffolding model with the monoploid genome selected by Khaper^15^ as a reference to identify allelic contigs. Finally, two haplotypes (HA and HB) were fully resolved at the chromosomal level.

To annotate protein-coding genes, we applied the same method as described previously for the sugarcane genome^41^. Briefly, we integrated evidence from orthologous proteins, transcriptomes and ab initio gene prediction using the MAKER pipeline^42^. In addition, we used RepeatMasker^43^ and TEclass^44^ to annotate repetitive sequences. GO and KEGG enrichment analyses of selected gene models were conducted with the OmicShare platform (www.omicshare.com/tools). Significance of enrichment was determined using Fisher’s exact test, with *P* values adjusted using the Benjamini–Hochberg multiple-hypothesis-testing correction.

### Estimation of switch errors in the phased assembly

A switch error indicates that a single base that is supposed to be present in one haplotype is incorrectly anchored onto another. This kind of assembly error is likely prevalent in the haplotype-resolved genome assembly. To detect switch errors in our phased chromosome-scale TGY genome assembly, we developed a new pipeline (calc_switchErr^19^), relying on a ‘true’ phased SNP dataset, which can be generated by incorporating PacBio long reads and 10x Genomics linked reads. The concept of the ‘true’ phased SNP dataset is to find consistently phased SNPs in PacBio read phasing and 10x read phasing. To achieve this, we first constructed an accurate variant-calling file (VCF) based on Illumina WGS short reads following the GATK^45^ best practices workflow suggested on the official website. Subsequently, approximately 80 Gb of PacBio long reads with length >10 kb were randomly selected and mapped against the reference genome using minimap2 (ref. ^46^) with the parameter ‘--secondary=no’, which means that only the best alignment was reported for each long read. The resulting BAM file along with the Illumina VCF was subjected to WhatsHap (version 1.1) phasing^47^ with default parameters, and the phased SNPs with the ‘PS’ label were extracted for further comparison. For phasing of 10x Genomics linked reads, we used proc10xG Python scripts (https://github.com/ucdavis-bioinformatics/proc10xG) to extract and trim reads of gem barcode information and primer sequences, respectively. This pipeline used BWA MEM for 10x linked reads mapping, and the resulting BAM file was also subjected to WhatsHap SNP phasing. Consistently phased SNPs in the two datasets were considered as ‘true’ phased SNPs, which were further used for assessment of ALLHiC phasing.

We next aligned two haplotypes in our ALLHiC assembly using the Nucmer program^48^ with parameters ‘--mum -l 100 -c 200 -g 200’, and variants were identified using show-snps with parameters ‘-Clr’, representing signatures of ALLHiC phasing. Subsequently, we compared ALLHiC phasing with the ‘true’ phased SNP dataset and identified switched bases if ALLHiC phased SNPs were inconsistent with the ‘true’ dataset. The pipeline with details of command lines is provided on GitHub (https://github.com/tangerzhang/calc_switchErr/).

### Identification of allelic variations and ASEGs

#### Identification of alleles

We used the same method as we did for an autopolyploid sugarcane genome project to identify alleles^41^. Because haplotype-resolved genome assembly is available for the TGY genome, each allele can be annotated from DNA sequences. The allele definition can be achieved using a synteny-based strategy and a coordinate-based method. Synteny blocks between two haplotypes were identified using MCScanX^49^, and paired genes within each synteny block with high similarity were considered as alleles A and B. Gene models with exactly the same coding sequences were considered as a single allele. In addition, gene models that were not present in syntenic blocks were mapped against the monoploid assembly using GMAP^50^. Potential alleles were considered if two genes had more than 50% overlap on coordinates.

#### Analysis of allelic variations at the gene level

We used the MAFFT program^51^ for pairwise comparison of allelic genes with default parameters. The edit distance between two alleles was counted if any base substitution or indel was detected using the Text Levenshtein distance model, implemented in PERL. The similarity score was calculated as the number of unsubstituted bases divided by the length of the alignment block.

#### Analysis of haplotype variations at the genome level

Pairwise comparison between haplotypes was performed using LAST version 959 (ref. ^52^), using the ‘NEAR’ seeding scheme, which favors short and strong similarities that are assumed to occur between closely related sequences. Haplotype A for each chromosome was used as input ‘as is’, with no external repeat masking except for simple repeats using tantan^53^ (lastdb parameters ‘-P0 -uNEAR -R01’). LAST alignments were then performed with lastal parameters ‘-E0.05 -C2’, followed by splitting alignments into one-to-one matches using last-split^54^. LAST alignments resulted in one MAF file that contained all high-scoring segment pairs per pairwise chromosome comparison. These resulting high-scoring segment pairs form the basis for calculating sequence identities in each pairwise comparison. Identities between haplotypes were calculated based on 10-Mb non-overlapping windows at the most stringent level with no indels or gaps within an alignment block. To identify different types of genetic variations between haplotypes, the Nucmer^48^ program was used to map HB to HA genomic sequences, and SNPs were identified from the alignment file with ambiguous best matches. Furthermore, we applied Assemblytics^55^ to identify short indels (1–10 bp) and large structural variants on the basis of the alignments above.

#### Analysis of allelic-specific expression

RNA-seq reads from six tissues (root, stem, flower, bud, young leaves and mature leaves) were generated using three biological replicates. RNA-seq reads were trimmed using the Trimmomatic^56^ program and mapped against allele-aware annotated gene models using Bowtie^57^ with only the best alignment retained for each read. FPKM values were estimated using the RSEM program^58^, which was implemented in the Trinity package^59^. ASE was determined if the log fold change of FPKM values between two alleles was greater than 2 with *P* value <0.05 and false discovery rate <0.05. Two different ASE patterns were investigated in this study, including consistent ASE and direction-shifting ASE.

#### Functional annotation of differentially expressed genes

GO enrichment and KEGG pathway analysis were performed using OmicShare tools (www.omicshare.com/tools). All functional enrichment analyses were calculated against a background gene set (that is, all predicted genes in the TGY genome), and background genes were submitted to the Mendeley database (10.17632/9nr63jfhtd.1) along with a functional annotation.

### Population genomics

#### Variant calling

We sequenced a total of 7.2 Tb of paired-end reads on the Illumina NovoSeq platform. This resulted in an average coverage of 12.75× per accession. To avoid potential DNA contamination, such as index swapping, we constructed dual-indexed libraries with unique indices for each sample. Double indices contain a total of 16 bases and were inserted in the flanking regions of the target DNA fragments. This allowed us to unambiguously separate DNA sequences pooled from different libraries and avoided potential index hopping. In addition, raw reads that had any mismatch with index sequences were clustered as undetermined sequences and finally removed from our analysis. Adaptors and low-quality bases (*Q* < 30) were trimmed from raw reads using Trimmomatic^56^, and the resulting clean reads were aligned against the monoploid reference genome of TGY using BWA^37^ with default parameters. To analyze population genetics, we focused on SNPs and small indels (1–10 bp). These variants were identified using the GATK^45^ pipeline following the best practices workflow suggested on the official website. To remove erroneous mismatches around small indels, IndelRealigner was applied to process the alignment BAM files. HaplotypeCaller and GenotypeCaller were used to call variants from all samples. SNPs were subjected to quality control and removed if they met the following criteria: (1) SNPs only present in one of the two datasets (HaplotypeCaller and GenotypeCaller), (2) SNPs in repeat regions, (3) SNPs with read depth >1,000 or <5, (4) SNPs with missing rate >40%, (5) SNPs with <5-bp distance from nearby variant sites, (6) non-biallelic SNPs. The SnpEff^60^ program was used to annotate SNPs and large-effect SNPs with modification of start or stop codon, and alternative splice sites were extracted for further analysis. SNP accuracy was assessed based on manual checking of 100 randomly selected SNPs in JBrowse^61^, showing an accuracy of 95%.

#### Maximum-likelihood tree inference

The high-quality SNPs identified above were subjected to a second round of filtering to improve the accuracy and efficiency of phylogenetic analysis. We first identified single-copy genes in the TGY genome based on a self-BLAST approach. Annotated coding sequences were subjected to all-versus-all self-BLAST alignment with default parameters, and the genes that only had one single BLAST hit (that is, self-match) were considered single-copy genes. A total of 11,334 single-copy genes were identified based on our method. Nuclear SNPs were further extracted from genomic regions located in single-copy genic regions. For heterozygous SNP sites, the major alleles were determined and retained for further analysis if they had more Illumina reads supported than the secondary alleles. The resulting SNPs were converted to aligned FASTA format. Maximum-likelihood trees were constructed using two popular programs: IQ-TREE^62^ with self-estimated best substitution models and RAxML^63^ with the GTRCAT model. The two phylogenetic trees were reconstructed based on 1,000 bootstrapping replicates, showing similar topology structures from the two programs.

#### Admixture analysis

Admixture^22^ software was used to infer the ancestral population among the resequenced tea accessions with different *k* values (from 1 to 10) tested. To avoid parameter standard errors, we allowed testing with 2,000 bootstraps. The optimal ancestral population structure was determined based on cross-validation error, with *k* = 7 showing the smallest cross-validation error and thus considered to be the best population size.

#### PCA, diversity statistics and linkage disequilibrium decay estimation

PLINK1.9 and VCFtools^64^ version 0.1.16 were used to perform PCA and other population divergency statistics, including nucleotide diversity and genetic differentiation (*F*_ST_). Linkage disequilibrium decay was calculated using PopLDdecay (version 3.31; https://github.com/BGI-shenzhen/PopLDdecay) with default parameters, and the decay distance of linkage disequilibrium indicates the Pearson’s correlation efficient (*r*^2^) decreased to half of the maximum.

#### Demographic analysis

We first calculated site-frequency spectrum (SFS) using ANGSD^65^. BAM files generated from each accession were filtered with parameters ‘-only_proper_pairs 1 -uniqueOnly 1 -remove_bads 1 -minQ 20 -minMap 30’. After that, we used the ‘-doSaf’ parameter to calculate the site allele-frequency likelihood based on individual genotype likelihoods, assuming HWE, and then used the realSFS with expectation–maximization algorithm to obtain a maximum-likelihood estimate of the folded SFS. The stairway plot^66^ was used for estimating the population demography history. Stairway plot was performed with 200 bootstraps, a generation time of 3 years and a mutation rate per generation per site of 6.5 × 10^−9^.

### Inference of selective sweeps

Patterns of selective sweeps associated with artificial selection were investigated based on three genetic differentiation metrics, including XP-EHH^67^ and Tajima’s *D*-test as well as population fixation statistics (*F*_ST_). To avoid false positive signals, we first filtered 26,318,206 of 35,725,355 (73.7%) SNPs located at TE regions, 12,030 of 35,725,355 (0.03%) SNPs at NUMT regions and 4,496 of 35,725,355 (0.01%) SNPs at NUPT regions before sweep finding. Subsequently, we applied the XP-EHH approach to identify positive selection sites by measuring cross-population extended haplotype homozygosity, which was implemented in the selscan program (https://github.com/szpiech/selscan). The XP-EHH score for each chromosome was calculated individually, and the top 5% sites with positive XP-EHH values were considered as signals for candidate selective sweeps. These candidate selective sweeps were further validated using Tajima’s *D* statistic and *F*_ST_ analysis. Tajima’s *D* statistic was calculated in sliding windows with a 10-kb window size and a 5-kb step size using the ANGSD program^65^, and the empirical lowest 5% windows were retained for validation of the candidate selective sweeps identified by XP-EHH. Similarly, *F*_ST_ values were calculated in VCFtools using the same sliding window size, and the top 5% regions were retained. XP-EHH candidate regions either supported by Tajima’s *D* statistic or the *F*_ST_ value between two tested populations were considered as the final set of selective sweeps.

### Identification of introgressed loci

#### *f*_3_ analysis

To detect introgression between cultivated tea plants and close relatives, we calculated *f*_3_ values using the program ADMIXTOOLS^22^; *Z* scores were adjusted based on a Benjamini–Hochberg false discovery-rate correction method.

#### ABBA–BABA analysis

To detect introgression between cultivated tea plants and close relatives, we calculated the Patterson’s *D* statistic using the program doAbbababa2, implemented in ANGSD^62^. Patterson’s *D* statistic is widely used to examine site patterns (also known as ABBA–BABA patterns^68^) in genome alignments for a specified four-taxon tree. Given four taxa with the relationship ‘((P1, P2), P3), O’, *a D* statistic significantly different from zero indicates introgression between populations P1 and P3 (negative *D* value) or between P2 and P3 (positive *D* value)^69^.

#### Modified *f*_d_ statistics

Introgressed loci were identified based on the modified four-taxon *f*_d_ statistics^22^, which is a modified version of a statistic originally developed to evaluate admixture at a genome-wide level. *C. oleifera* was used as an outgroup to infer phylogeny of the tested triplets (P1, P2 and P3), with a combination of any of the four cultivated tea populations (P2) and close relatives from *Camellia* section *Thea* (P1 or P3). Modified *f*_d_ statistics were calculated for each 100-kb non-overlapping window with the high-quality of SNP data identified above as input using a set of Python scripts (https://github.com/simonhmartin/genomics_general/blob/master/ABBABABAwindows.py). Windows with a negative Patterson’s *D* statistic and *f*_d_ > 1 were ignored as suggested^24^. Within each cultivated tea population, we used a threshold of the 95th percentile to detect outliers of the *f*_d_ distribution that could be considered as introgressed loci from close relatives.

### Reporting Summary

Further information on research design is available in the Nature Research Reporting Summary linked to this article.

## Online content

Any methods, additional references, Nature Research reporting summaries, source data, extended data, supplementary information, acknowledgements, peer review information; details of author contributions and competing interests; and statements of data and code availability are available at 10.1038/s41588-021-00895-y.

## Supplementary information

Supplementary Information Supplementary Notes 1 and 2, Figs. 1–13 and Tables 1–13 and 15–17

Reporting Summary

Supplementary Table 14 Information and statistics of resequenced tea accessions.

## Figures and Tables

**Fig. ExtendedData 4:**
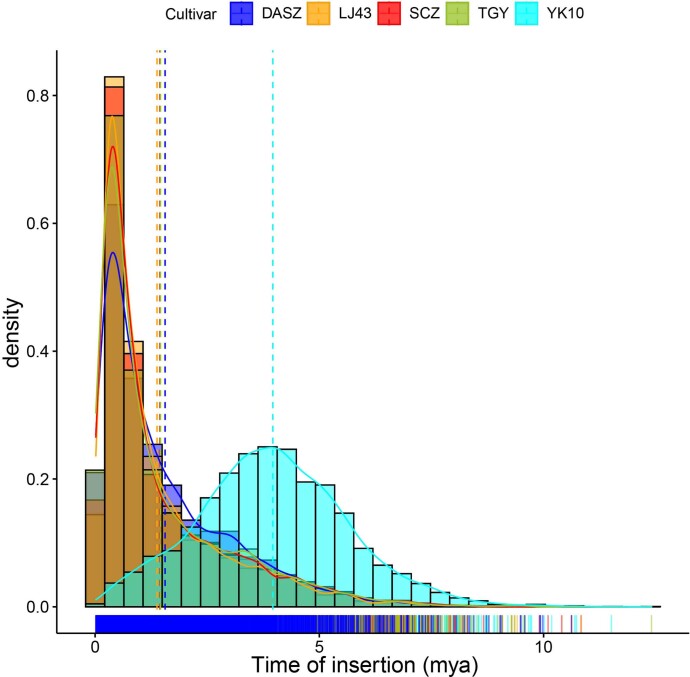
**Estimation of the LTR burst time based on intact LTRs identified by LTR_retriever**.

## Data Availability

Raw sequencing reads from PacBio, Illumina, 10× Genomics, Hi-C, RNA-seq and Iso-seq were deposited in the National Center for Biotechnology Information database under the accession number PRJNA665594 and/or in the GSA database (https://bigd.big.ac.cn/gsa/) under the accession number PRJCA003090. The assembly and annotation were archived in the National Center for Biotechnology Information under the accession number JAFLEL000000000 and in the GWH (https://bigd.big.ac.cn/gwh/) under accession numbers GWHASIV00000000 for the monoploid and GWHASIX00000000 for the haplotype-resolved genome. VCF files that contain all clean SNPs were uploaded to the Mendeley database (https://data.mendeley.com/datasets/7hb33vd7sf/1). In addition, three datasets that were used to assess switch errors in the haplotype-resolved TGY genome assembly were deposited to the Mendeley database (10.17632/xpccyg5w2x.1).

## Code availability

The Khaper algorithm is freely available at GitHub (https://github.com/lardo/khaper), and calc_switchErr can be found on GitHub (https://github.com/tangerzhang/calc_switchErr/). Codes (Khaper and calc_switchErr) were also archived on Zenodo with the DOIs 10.5281/zenodo.4780792 and 10.5281/zenodo.4780666 and are cited in refs. ^15^^,^^19^.
