# Supplementary material for: Haplotype-resolved genome assembly provides insights into evolutionary history of the tea plant Camellia sinensis
Source: Nat Genet. 2021 Jul 15;53(8):1250–9. doi: 10.1038/s41588-021-00895-y (PMC8346365; doi:10.1038/s41588-021-00895-y)
Supplement: Supplementary file 1 — Supplementary Notes 1 and 2, Figs. 1–13 and Tables 1–13 and 15–17 [file 41588_2021_895_MOESM1_ESM.pdf]

nature portfolio

https://doi.org/10.1038/s41588-021-00895-y

Supplementary information

Haplotype-resolved genome assembly provides insights into evolutionary history of the tea plant *Camellia sinensis*

In the format provided by the authors and unedited

Haplotype-resolved genome assembly provides insights into evolutionary history of the tea plant *Camellia sinensis*

Xingtan Zhang[1,2,†,*], Shuai Chen[1,3,4,†], Longqing Shi[1,3,5,†], Daping Gong[6,†], Shengcheng Zhang[4], Qian Zhao[1,5], Dongliang Zhan[7], Liette Vasseur[1,5,8], Yibin Wang[4], Jiaxin Yu[4], Zhenyang Liao[4], Xindan Xu[4], Rui Qi[4], Wenling Wang[4], Yunran Ma[4], Pengjie Wang[9], Naixing Ye[9], Dongna Ma[1], Yan Shi[1], Haifeng Wang[1], Xiaokai Ma[4], Xiangrui Kong[10], Jing Lin[4], Liufeng Wei[1], Yaying Ma[4], Ruoyu Li[4], Guiping Hu[1,11], Haifang He[1], Lin Zhang[12], Ray Ming[13], Gang Wang[14] ,Haibao Tang[4,*], Minsheng You[1,5,*]

[1]State Key Laboratory of Ecological Pest Control for Fujian and Taiwan Crops, Institute of Applied Ecology, College of Plant Protection, Fujian Agriculture and Forestry University, Fuzhou 350002, China

[2]Shenzhen Branch, Guangdong Laboratory for Lingnan Modern Agriculture, Genome Analysis Laboratory of the Ministry of Agriculture, Agricultural Genomics Institute at Shenzhen, Chinese Academy of Agricultural Sciences, Shenzhen, 518120, China

[3]Institute of Rice, Fujian Academy of Agricultural Sciences, Cangshan, Fuzhou 350018, China

[4]Center for Genomics and Biotechnology, Fujian Provincial Key Laboratory of Haixia Applied Plant Systems Biology, Key Laboratory of Genetics, Fujian Agriculture and Forestry University, Fuzhou 350002, China

[5]Joint International Research Laboratory of Ecological Pest Control, Ministry of Education, Fuzhou 350002, China

[6]Tobacco Research Institute of Chinese Academy of Agricultural Sciences, Qingdao, 266101, China

[7]Hangzhou Kaitai Biotech Co. Ltd, Hangzhou, 310000, China

[8]Department of Biological Sciences, Brock University, 1812 Sir Isaac Brock Way, St. Catharines, ON L2S 3A1, Canada (ORCID #0000-0001-7289-2675)

[9]Key Laboratory of Tea Science, College of Horticulture, Fujian Agriculture and Forestry University, Fuzhou 350002, China

[10]Tea research institute, Fujian Academy of Agricultural Sciences, No. 104, Pudang Road, Jinan District, Fuzhou 350003, China

[11]Jiangxi Sericulture and Tea Research Institute, Nanchang County, Nanchang, 330202 China

[12]Key Laboratory of Cultivation and Protection for Non-Wood Forest Trees, Ministry of Education, Central South University of Forestry and Technology, Changsha 410004, China

[13]Department of Plant Biology, University of Illinois at Urbana-Champaign, Urbana, Illinois 6180, USA

[14]CAS Key Laboratory of Tropical Forest Ecology, Xishuangbanna Tropical Botanical Garden, Chinese Academy of Sciences, Mengla 666303, China

†These authors contributed equally to this work: Xingtan Zhang, Shuai Chen, Longqing Shi, Daping Gong

*Correspondence, Email: zhangxt@fafu.edu.cn; msyou@fafu.edu.cn; tanghaibao@gmail.com

Supplementary Notes

Supplementary Note 1. Development of Khaper: a kmer-based method to identify and retain representative haplotype for a heterozygous diploid genome

Motivation. Assembly of the heterozygous diploid genome usually involves in removing redundant sequences that are likely originated from allelic contigs. Currently, to our knowledge, there are three strategies that are capable of filtering the redundant contigs from initial contig assemblies, including read-depth (RD), whole genome alignment comparison (WGAC) and Kmer-based. The RD approach, with purge_haplotigs [1] as a successful example, investigates the read depth across the initial contigs through mapping raw sequencing data against the reference assembly. For a heterozygous diploid genome, plotting the read depth of these contigs shows a bimodal distribution. Contigs with 1x coverage of sequencing reads indicate that they are haplotype-fused or collapsed assembly of the genome; while, contigs with ~0.5x coverage are haplotype resolved sequences with presence of heterozygous allelic sequences. Therefore, the basic concept of purge_haplotigs is to identify and remove alternative allelic contigs based on distribution of read depth. The second strategy to filter heterozygous sequences is based on whole genome alignment comparison, for instance Pseudohaploid [2] . This algorithm starts by aligning the genome assembly against itself and pairwise comparison of allelic contigs will lead to long "alignment chains", indicating redundant homologous regions. One copy within the homologous regions will be retained as a representative haplotype. Both of the two strategies are efficient to solve heterozygous diploid with moderate genome size (2 Gb or less), however, processing large genomes will cost much CPU time and computational resources as alignment of DNA sequences at whole genome level is time-consuming.

Overview of Khaper. The Kmer-based approach showed its efficiency to separate haplotypic sequences even from a large amount of data with hundreds of Gb size. For instance, the recently developed PacBio assembler, CANU trio-binning [3] , utilized the Illumina sequencing reads from parental genomes to separate the PacBio long reads into two categories, presenting phased genomic sequences from parents. Additionally, our previous study showed that the Kmer-based approach was able to solve the problem of the highly heterozygous moth genome assembly, by developing a program called Rabbit[4]. However, to our knowledge, there is no software that can directly implement the Kmer-based approach to identify allelic contigs from the initial contig assembly. To develop an efficient tool to remove redundant sequences for a large genome such as C. sinensis, we propose a program – Khaper (Kmer-based haplotype caller; Supplementary Figure 1). Khaper implemented the core algorithm from Rabbit [4] but with two major differences. Khaper is designed for removing redundant sequences from PacBio

assemblies and therefore is able to take either Illumina short reads or PacBio long reads as input. Meanwhile, Khaper retained and re-compiled the redundancy reducing function of Rabbit using C++ with much improved speed, making it broadly applicable to a wild range of genome projects. Khaper starts from a genome survey using 17-mers extracted from Illumina short reads or PacBio long reads by Jellyfish [5]. Investigation of 17-mers reveals a bimodal distribution of Kmer depth. Ideally, the first peak located at 1/2 coverage of the second peak, which represent heterozygous peak and homozygous peak (main peak), respectively. We set 1.5 × depth of the main peak as a cutoff, and categorized K-mers as "Non-Repeat K-mers" and "Repeat K-mers" as illustrated in Supplementary Figure 1b. Non-Repeat K-mers were tracked back to genome assembly and non-repeat regions were identified for each contig (Supplementary Figure 1c). Further, pairwise comparison between contigs identify primary contigs and redundant sequences if they share a large proportion of Non-Repeat regions (for example 40% in our case). The contigs with longer size are retained as a representative haplotype (Supplementary Figure 1d).

Comparison of Khaper with related programs. We compared Khaper with the other two state-of-art programs, Purge_haplotigs and Pseudohaploid, which represent RD and WGAC strategies. The data sets we generated for C. sinensis de novo assembly was used for program testing, including the CANU initial assembly (5.4 Gb), 58× illumina reads and 114× Pacbio long reads. We tested these data sets on a 28-core Linux server with 128 Gb RAM and allow the tested programs to allocate cores as many as possible. Other parameters were kept as default (see below for command line details). Comparison of these programs reveal that Khaper consumed 1,194 minutes for CPU time and 390 minutes for real time, at least 3.7 and 6.8 times faster than other programs, respectively (Supplementary Table 1). In addition, Khaper generated a reasonable genome assembly (3.06 Gb) after filter redundant sequences, however, assembly sizes in other programs were larger than estimated genome size (3.15 Gb). Meanwhile, we observed that contig N50 in Khaper was the most optimal one among all of the test data. The BUSCO completeness and duplication score in Khaper were comparable with outputs from other programs. Taken together, our newly developed program, Khaper, is fast and efficient to remove redundant sequences for a highly heterozygous diploid species with large genome size.

Command lines of Khaper, Purge_haplotigs and Pseudohaploid used for testing.

(1) Khaper

$ perl Graph.pl pipe -i fq.list -m 2 -k 17 -s 1,3 -d Kmer_17

$ perl remDup.pl --kbit Kmer_17/02.Uinque_bit/kmer_17.bit --kmer 17 genome.fa RemDup 0.4

(2) Purge_haplotigs for Pacbio subreads

```
$ minimap2 -ax map-pb -t 28 genome.fa pb.merge.fasta.gz --secondary=no --split-prefix ref \
        | samtools sort -@ 20 -m 1G -o aligned.bam -T tmp.ali
        | samtools sort -@ 12 -m 1G -o aligned.bam -T tmp.ali
$ purge_haplotigs hist -t 28 -b aligned.bam -g genome.fa
$ purge_haplotigs cov -i aligned.bam.gencov -l 10 -m 85 -h 130
$ purge_haplotigs purge -g genome.fa -c coverage_stats.csv -t 28 -o purge
```

(3) Purge_haplotigs for Illumina short reads

```
$ bwa index genome.fa
$ samtools faidx genome.fa
$ bwa mem -t 28 genome.fa zwt_R1.fq.gz zwt_R2.fq.gz \
   | /public/home/tanger/software/samtools-1.3/samtools view -hF 256 - \
   | /public/home/tanger/software/samtools-1.3/samtools sort -@ 12 -m 4G -o aligned.bam -T tmp.ali
$ samtools index aligned.bam
$ purge_haplotigs hist -t 28 -b aligned.bam -g genome.fa
$ purge_haplotigs cov -i aligned.bam.gencov -l 10 -m 85 -h 130
$ purge_haplotigs purge -g genome.fa -c coverage_stats.csv -t 28 -o purge
```

(4) Pseudohaploid

```
$ ./create_pseudohaploid.sh draft.asm.fasta clean MIN_IDENTITY=90 MIN_LENGTH=1000 MIN_CONTAIN=93 MAX_CHAIN_GAP=20000
```

Supplementary Note 2. Genome sequencing and assembly of C. sinensis cultivar 'Tieguanyin' Genome

Illumina short reads sequencing. For the genome sequencing of TGY, we collected the leaf samples from the same individual and extracted DNA using Qiagen DNeasy Plant Mini Kit. The DNA library was constructed by selecting fragments with length ranging from 300-500 bp, i.e., insert size 300-500. Afterwards, we sequenced the DNA library on Illumina NovaSeq platform with 150-bp PE (paired-end) model.

Placbio library construction and Sequencing. The extracted DNA aforementioned was sheared, concentrated and further applied to size-selection by BluePippin system according to the manufacturer's instruction. We constructed ~ 20 kb SMRTbell^{TM} libraries and a total of three Single-Molecule Real-Time (SMRT) cells were sequenced on Pacbio Sequel II platform, generating 359 Gb of subreads (Table 1).

Hi-C library construction and sequencing. The tender leaves collected from the same individual that was used for genome sequencing were subjected to construction of Hi-C

libraries according to the method described before[6]. MboI was used to digest the cross-linked DNA over-night and biotins were added to the end of fragmented DNA sequences. The chimeric junctions formed by proximity ligation were enriched by extracting biotins and further physically sheared, generating DNA fragments with 500-700 bp size. We sequenced these DNA fragments on Illumina NovaSeq platform with PE model. A total of 1,038 million of 150-bp paired-end reads were produced and the quality was assessed using HiC-Pro program[7], showing 72.1% of validate Hi-C reads (Supplementary Table 2).

Estimation of TGY genome size. We estimate the nuclear DNA content based on flow cytometry, showing the genome size for a haploid or 1C is 3.15 Gb, close to previously published tea genomes [8–11].

Contig assembly. We corrected, trimmed and assembled the full PacBio reads using the CANU assembler version 1.9[12] with optimal parameters for polyploid genome phasing (batOptions=-dg 3 -db 3 -dr 1 -ca 500 -cp 50). The initial contig assembly resulted in a 5.4-Gb with a contig N50 of 925.7 kb. This assembly size accounts for 172% of estimated genome size (3.15Gb), indicating a large proportion of redundant sequences present in the draft genome. To remove redundant sequences, we used three programs, including Khaper, Purhaplotigs and Pseudohaploid (Supplementary Note 1). Results are assessed based on genome size, BUSCO scores as well as contig N50. Finally, the 3.06-Gb assembly generated by Khaper was selected for Hi-C scaffolding. The Illumina short reads were further used to polish contig assembly, implemented in the Pilon program[13].

Hi-C scaffolding and chromosome assembly for a monoploid genome. We first mapped the Hi-C reads against the contig assemblies and detected mis-joined assembly by searching for abnormal long-rang contact patterns in 3D-DNA pipeline[14]. The 3D-DNA pipeline performs iterative scaffolding with several rounds of correction. To avoid the assembly errors introduced by the iterative scaffolding steps, we limited only the first round of Hi-C correction in the 3D-DNA pipeline (i.e., only correction of contigs rather than correction of scaffolds) in our improved haplotype-resolved TGY genome assembly. The Hi-C corrected contigs were further suggested to ALLHiC scaffolding[15], resulting in a chromosome-scale assembly with 15 pseudo-chromosomes anchored. We assessed the accuracy of Hi-C assembly by chromatin contact matrix (Extended Data Fig. 1b).

Haplotype-resolved chromosomal level assembly. The CANU initial contig assembly was used for haplotype-resolved chromosomal level assembly. To rescue collapsed regions, 184 Gb whole genome shotgun reads sequenced by Illumina Nova-seq platform were mapped against the CANU assembly and copy number was calculated for each contig

using a home-make PYTHON script. Ideally, a phased contig (i.e., both of allelic contigs are present in assembly) will have one copy in the draft genome assembly, while a haplotype-fused contig has two copies. The haplotype-fused contigs were duplicated and subject to Hi-C scaffolding along with haplotype phased contigs. The modified contig assembly resulted in 5.80 Gb genome sequences and were linked into 30 phased chromosomes in ALLHiC pipeline with polyploid model. After that, we manually corrected assembly errors, especially chimeric scaffolds, based on synteny analysis between the haplotype-resolved assembly and the monoploid chromosomal level assembly. Finally, a haplotype-resolved chromosomal level of C. sinensis cultivar TGY genome was released.

Validation of genome assembly. We assessed the assembly completeness based on 1,375 conserved plant genes in BUSCO program[16] with default parameters. BUSCO reported 93.7% and 95.2% of completeness for the monoploid genome and the haplotype-resolved genome, respectively (Table 1 and Supplementary Table 1). In addition, Illumina reads were aligned to the monoploid assembly using BWA[17], revealing that 99.74% of reads were mappable (Supplementary Table 5), covering 98.6% of TGY genome sequences. These results indicate a high level of assembly completeness and accuracy in our assemblies (Supplementary Table 5). Compared to the recently published two chromosome-scale CSS assemblies, the TGY genome is superior in continuity (contig N50: 1.94 Mb vs. 0.6 Mb for CSS-SCZ and 0.27 Mb for CSS-LJ43) and BUSCO completeness (93.7% vs.90.6% for CSS-SCZ and 90.0% for CSS-LJ43), though the statistics are slightly lower than the assembly of wild tea plant, CSA-DASZ (a contig N50 of 2.59 Mb and BUSCO completeness of 95.1%; **Supplementary Table 4**). Synteny analysis between the TGY genome with CSS or CSA revealed high consistency and a number of genomic rearrangements were detected (**Extended Data Figs. 2-3**). Assessment using LTR Assembly Index (LAI)[18] revealed more intact LTRs in the TGY genome, qualifying it as a reference genome (**Supplementary Table 4 and Extended Data Fig. 1c**).

References

1. Roach, M. J., Schmidt, S. A. & Borneman, A. R. Purge Haplotigs: allelic contig reassignment for third-gen diploid genome assemblies. *BMC Bioinformatics* **19**, 460 (2018).
2. Chen, L.-Y. *et al.* The bracteatus pineapple genome and domestication of clonally propagated crops. *Nature Genetics* **51**, 1549–1558 (2019).
3. Koren, S. *et al.* De novo assembly of haplotype-resolved genomes with trio binning.

Nature Biotechnology **36**, 1174–1182 (2018).
4. You, M. *et al.* A heterozygous moth genome provides insights into herbivory and detoxification. *Nature Genetics* **45**, 220–225 (2013).
5. Marçais, G. & Kingsford, C. A fast, lock-free approach for efficient parallel counting of occurrences of k-mers. *Bioinformatics* **27**, 764–770 (2011).
6. Xie, T. *et al.* De novo plant genome assembly based on chromatin interactions: a case study of Arabidopsis thaliana. *Mol Plant* **8**, 489–492 (2015).
7. Servant, N. *et al.* HiC-Pro: an optimized and flexible pipeline for Hi-C data processing. *Genome Biology* **16**, 259 (2015).
8. Zhang, W. *et al.* Genome assembly of wild tea tree DASZ reveals pedigree and selection history of tea varieties. *Nature Communications* **11**, 3719 (2020).
9. Xia, E.-H. *et al.* The Tea Tree Genome Provides Insights into Tea Flavor and Independent Evolution of Caffeine Biosynthesis. *Molecular Plant* **10**, 866–877 (2017).
10. Wang, X. *et al.* Population sequencing enhances understanding of tea plant evolution. *Nature Communications* **11**, 4447 (2020).
11. Wei, C. *et al.* Draft genome sequence of Camellia sinensis var. sinensis provides insights into the evolution of the tea genome and tea quality. *Proceedings of the National Academy of Sciences* **115**, E4151–E4158 (2018).
12. Koren, S. *et al.* Canu: scalable and accurate long-read assembly via adaptive k -mer weighting and repeat separation. *Genome Research* **27**, 722–736 (2017).
13. Walker, B. J. *et al.* Pilon: An integrated tool for comprehensive microbial variant detection and genome assembly improvement. *PLoS ONE* **9**, e112963 (2014).
14. Dudchenko, O. *et al.* De novo assembly of the Aedes aegypti genome using Hi-C yields chromosome-length scaffolds. *Science* **356**, 92–95 (2017).
15. Zhang, X., Zhang, S., Zhao, Q., Ming, R. & Tang, H. Assembly of allele-aware, chromosomal-scale autopolyploid genomes based on Hi-C data. *Nature Plants* **5**, (2019).
16. Simão, F. A., Waterhouse, R. M., Ioannidis, P., Kriventseva, E. v & Zdobnov, E. M. BUSCO: assessing genome assembly and annotation completeness with single-copy orthologs. *Bioinformatics* **31**, 3210–3212 (2015).
17. Li, H. & Durbin, R. Fast and accurate short read alignment with Burrows-Wheeler transform. *Bioinformatics* **25**, 1754–1760 (2009).
18. Ou, S., Chen, J. & Jiang, N. Assessing genome assembly quality using the LTR Assembly Index (LAI). *Nucleic Acids Res.* (2018) doi:10.1093/nar/gky730.

Supplementary Table 1. Comparison of our newly developed algorithm (Khaper) with the programs of Purge_haplotigs and Pseudohaploid

	Input assembly (canu initial assembly)	Khaper	Purge_haplotigs		Pseudohaploid
Strategy		K-mer	Read depth		Whole genome alignment
Data set	N.A	Draft assembly/Illumina reads (58 ×)	Draft assembly/Pacbio reads (114 ×)	Draft assembly/Illumina reads (58 ×)	Draft assembly
Real Time (min)	N.A.	390	1780	1452	7049
CPU Time (min)	N.A.	1194	40441	29016	8117
Assembly size (Gb)	5.41	3.06	3.26	3.27	4.99
BUSCO completeness (%)	95.6	93.7	95.2	95.5	95.7
BUSCO duplication (%)	70.0	10.1	9.6	22.8	60.0
Contig N50 (Mb)	0.93	1.94	1.78	1.77	1.07
No. of contigs	17,662	3699	4941	5156	11803

N.A. indicates Not Available.

Supplementary Table 2. Statistics of Hi-C mapping of the 'TGY' genome

Statistics of mapping	
Clean Paired-end Reads	1038167101
Unmapped Paired-end Reads	28846838
Unmapped Paired-end Reads Rate (%)	2.777
Paired-end Reads with Singleton	173880209
Paired-end Reads with Singleton Rate(%)	16.74
Multi Mapped Paired-end Reads	475187581
Multi Mapped Ratio (%)	45.79
Unique Mapped Paired-end Reads	360252473
Unique Mapped Ratio (%)	34.693
**Statistics of valid reads**	
Unique Mapped Paired-end Reads	360252473
Dangling End Paired-end Reads	24211205
Dangling End Rate (%)	6.721
Self Circle Paired-end Reads	6205678
Self Circle Rate (%)	1.723
Dumped Paired-end Reads	51006791
Dumped Rate (%)	14.159
Interaction Paired-end Reads	274949410
Interaction Rate (%)	76.321
Lib Valid Paired-end Reads	198170721
Lib Valid Rate (%)	72.1
Lib Dup (%)	27.9

Supplementary Table 3. Statistics of chromosomal level monoploid assembly in ‘TGY’

ChrID	No. of contigs	Length (bp)
Chr1	262	260823534
Chr2	618	263612798
Chr3	245	212239356
Chr4	240	229735363
Chr5	212	229482998
Chr6	276	236925330
Chr7	229	214919478
Chr8	174	213467978
Chr9	188	170688365
Chr10	190	200071334
Chr11	170	189546957
Chr12	241	162252815
Chr13	220	167407016
Chr14	147	138816800
Chr15	139	141162172
Total No. of contigs		3699
Total length of contigs (Gb)		3.06
Total No. of anchored contigs		3551
Total length of chromosome level assembly (Mb)		3.03
Anchor rate (%)		98.96

Supplementary Table 4. Comparison of contig assemblies among five genomes of tea accessions

	CSS (TGY)		CSA (Yunkang10)	CSS-SCZ	CSA-DASZ	CSS-LJ43
	Initial contig assembly	Monoploid assembly				
No. Of contigs	17,662	3,699	37,618	7,031	5,453	37,600
Max length (Mb)	13.71	13.71	3.5058	2.88	16.83	2.43
Assembly size (Mb)	5,410.47	3,062.53	3,021.23	2,938.18	3099	3260
Contig N90 (bp)	107,813	413,666	83,917	209,326	393,931	35,684
Contig N50 (bp)	925,696	1,941,180	449,457	600,461	2,589,771	271,330
Average (bp)	306,333	827,933	80,313	417,864	574,796	35,684
Complete BUSCO ratio (%)	95.4%	93.7%	90.2%	90.6%	95.1	90.0
Raw LAI	10.04	8.48	1.70	8.63	8.29	7.35
LAI	10.00	10.17	2.05	8.14	10.08	9.58

Supplementary Table 5. Assessment of monoploid genome consistency based on Illumina reads

Item	Statistic
Number of reads	612,510,318
Data size (Gb)	91.87
Mapped bases (Gb)	91.63
Mapping rate (%)	99.74
Genome Length (Mbp)	3062.53
Mean Depth	53.61
Coverage Rate (%)	98.6
Regions with low coverage (< 5 reads)	90,141,266
Percentage with low coverage (< 5 reads)	0.0508%
Number of homozygous variants	169,477
Percentage of homozygous variants	0.051%

Supplementary Table 6. BUSCO analysis of annotation completeness in TGY monoploid genome

Description	C. sinesis cultivar 'TGY'	
	Number	Percentage (%)
Complete BUSCOs(C)	1266	92.1
Complete and single-copy BUSCOs(S)	1151	83.7
Complete and duplicated BUSCOs(D)	115	8.4
Fragmented BUSCOs(F)	31	2.3
Missing BUSCOs(M)	78	5.6
Total BUSCO groups searched	1375	100.0

Supplementary Table 7. TE annotation of three tea genomes

	CSA-YK10		CSS(Shuchazao)		CSS(TGY)	
	Length(Mb)	% of genome	Length(Mb)	% of genome	Length(Mb)	% of genome
Total repeat fraction	1911.91	63.28	2071.55	65.94	2391.46	78.15
Class I: Retroelement	1685.23	55.78	1764.84	56.18	1957.15	63.96
LTR Retrotransposon	1312.97	43.46	1385.35	44.1	1587.84	51.89
Ty1/Copia	112.85	3.74	135.22	4.3	147.82	4.83
Ty3/Gypsy	679.02	22.47	638.44	20.32	832.23	27.20
Other	521.11	17.25	611.70	19.47	607.79	19.86
Non-LTR retrotransposon	244.59	8.1	246.42	7.84	242.72	7.93
LINE	235.02	7.78	231.98	7.38	229.00	7.48
SINE	9.57	0.32	14.4	0.46	13.72	0.45
Unclassified retroelement	127.67	4.23	133.06	4.24	126.59	4.14
Class II: DNA transposon	318.70	10.55	434.13	13.82	642.80	21.01
TIR						
CMC[DTC]	20.27	0.67	20.72	0.66	24.29	0.79
hAT	40.87	1.35	46.73	1.49	56.52	1.85
Mutator	29.55	0.98	26.74	0.85	24.97	0.82
Tc1/Mariner	0.35	0.01	2.61	0.08	4.19	0.14
PIF/Harbinger	22.88	0.76	25.17	0.8	55.59	1.82
Other	204.44	6.77	309.56	9.85	473.04	15.46
Helitron	7.05	0.23	20.15	0.64	12.51	0.41
Tandem repeats	182.59	6.04	170.94	5.44	124.65	4.07
Unknown	60.28	2.00	74.29	2.36	88.62	2.90

Supplementary Table 8. Statistics of intact LTRs identified by LTR_retriever

Genome	Superfamily	TE type	Number of intact LTR	Total
	Gypsy	LTR	1,667	
CSA(Yunkang10)	Copia	LTR	679	3,041
	unknown	LTR	694	
	Gypsy	LTR	2,131	
CSS(Shuchazao)	Copia	LTR	1,285	4,718
	unknown	LTR	1,301	
	Gypsy	LTR	8,969	
CSS(TGY)	Copia	LTR	48,18	20,969
	unknown	LTR	7,181	

Supplementary Table 9. Haplotype-resolved chromosomal level assembly and annotation of TGY genome

ChrID	Haplotype A		Haplotype B	
	Length (Mb)	No. of allelic genes	Length (Mb)	No. of allelic genes
Chr1	261.6	3,016	277.1	2,288
Chr2	206.2	2,578	242.9	2,051
Chr3	229.7	2,437	208.4	1,882
Chr4	244.8	2,751	221.4	1,902
Chr5	214.0	2,332	181.1	1,721
Chr6	246.5	2,645	203.1	1,697
Chr7	260.7	2,461	198.6	1,345
Chr8	204.1	1,890	206.9	1,513
Chr9	208.5	2,304	175.8	1,759
Chr10	188.4	1,908	187.1	1,496
Chr11	137.1	1,500	155.5	1,477
Chr12	169.2	1,707	173.9	1,353
Chr13	1695	1,796	181.5	1,410
Chr14	177.2	1,847	179.3	1,598
Chr15	140.2	1,424	125.0	1,231
Total No. of contigs			60345	
Total length of contigs (Mb)			5987	
Total No. of anchored contgis			45045	
Total length of chromosome level assembly (Mb)			5975	
Anchor rate (%)			99.72	

Supplementary Table 10. Statistics of genetic variation between the two haplotypes in the 'TGY' genome

ChrID	No. of SNPs	No. of insertions	No. of deletions	ChrID	No. of SNPs	No. of insertions	No. of deletions
Chr01	346833	10441	10469	Chr09	241192	7017	7214
Chr02	281413	8606	8664	Chr10	238299	8618	8460
Chr03	251611	7521	7434	Chr11	166717	5403	5291
Chr04	274057	8753	8858	Chr12	251451	8209	8388
Chr05	324626	9722	9894	Chr13	184866	7449	7061
Chr06	246867	7582	7447	Chr14	335139	11419	11227
Chr07	174122	6194	6075	Chr15	185904	5829	5879
Chr08	195662	5937	5974	Total	3698759	118700	118335

Supplementary Table 11. TE annotation between the two haplotypes in the 'Tieguanyin' genome

	Haplotype A		Haplotype B	
	Length(Mb)	% of genome	Length(Mb)	% of genome
Total repeat fraction	2,269.42	74.28	2,162.81	74.19
Class I: Retroelement	1,890.03	61.86	1,804.57	61.90
LTR Retrotransposon	1,491.40	48.81	1,421.48	48.76
Ty1/Copia	118.79	3.89	114.16	3.92
Ty3/Gypsy	778.51	25.48	744.83	25.55
Other	594.10	19.44	562.50	19.29
Non-LTR retrotransposon	257.33	8.42	248.11	8.51
LINE	243.14	7.96	234.44	8.04
SINE	14.19	0.46	13.67	0.47
Unclassified retroelement	141.30	4.62	134.98	4.63
Class II: DNA transposon	415.44	13.60	394.95	13.55
TIR				
CMC[DTC]	9.26	0.30	9.26	0.32
hAT	10.67	0.35	9.98	0.34
Mutator	54.67	1.79	52.30	1.79
Tc1/Mariner				
PIF/Harbinger	3.64	0.12	3.28	0.11
Other	337.20	11.04	320.13	10.98
Helitron	15.41	0.50	14.97	0.51
Tandem repeats	61.52	2.01	56.15	1.93
Unknown	96.83	3.17	92.14	3.16

Supplementary Table 12. Number of genes showing biased expression toward haplotype A (i.e., A>B) or haplotype B (A<B).

	Stem		Bud		Root		Flower		Young leaf		Mature leaf	
	A>B	A<B	A>B	A<B	A>B	A<B	A>B	A<B	A>B	A<B	A>B	A<B
Chr01	208	221	192	213	187	207	160	198	216	214	191	197
Chr02	211	155	209	153	194	148	197	180	207	149	185	131
Chr03	153	160	138	162	133	153	119	174	158	156	143	136
Chr04	176	170	177	163	152	159	182	130	176	177	152	150
Chr05	149	143	139	137	142	137	158	116	142	144	135	126
Chr06	150	180	135	184	144	182	76	231	147	190	139	174
Chr07	123	116	116	121	105	122	102	86	125	121	112	121
Chr08	133	122	128	112	132	101	107	104	131	124	123	108
Chr09	222	141	224	128	232	133	195	127	221	136	205	128
Chr10	122	136	122	114	115	131	104	121	115	135	108	129
Chr11	97	139	80	126	84	126	78	140	88	140	87	129
Chr12	185	104	181	97	180	95	94	95	185	105	165	96
Chr13	124	108	116	108	116	108	100	125	124	106	110	98
Chr14	122	144	123	144	105	146	114	120	119	156	111	148
Chr15	108	113	88	107	75	108	40	155	101	114	94	103

* Designation of A and B between different chromosomes are arbitrary.

Supplementary Table 13. KEGG analysis of 386 inconsistent allele specifically expressed genes. P values were calculated using two-sided Fisher's exact test and further corrected based on Benjamini-Hochberg false discovery rate correction method.

#	Pathway	Candidate genes with pathway annotation (82)	All genes with pathway annotation (8052)	*P*value	Qvalue	Pathway ID
1	Glutathione metabolism	5 (6.1%)	161 (2%)	0.023865	0.705718	ko00480
2	alpha-Linolenic acid metabolism	3 (3.66%)	61 (0.76%)	0.023995	0.705718	ko00592
3	Flavone and flavonol biosynthesis	1 (1.22%)	3 (0.04%)	0.030245	0.705718	ko00944
4	Terpenoid backbone biosynthesis	3 (3.66%)	81 (1.01%)	0.049282	0.720923	ko00900
5	Flavonoid biosynthesis	3 (3.66%)	85 (1.06%)	0.055447	0.720923	ko00941
6	Glycosphingolipid biosynthesis - ganglio series	1 (1.22%)	7 (0.09%)	0.06917	0.720923	ko00604
7	Phenylpropanoid biosynthesis	5 (6.1%)	219 (2.72%)	0.072092	0.720923	ko00940
8	Stilbenoid, diarylheptanoid and gingerol biosynthesis	2 (2.44%)	52 (0.65%)	0.098051	0.765292	ko00945
9	Ether lipid metabolism	2 (2.44%)	54 (0.67%)	0.104473	0.765292	ko00565
10	Synthesis and degradation of ketone bodies	1 (1.22%)	12 (0.15%)	0.115663	0.765292	ko00072

Supplementary Table 15. Summary of the variants in different clustered groups of tea populations.

Type	Subgroups	Number of re-sequenced accessions	Ratio of non-synonymous to synonymous SNPs	π
CT	CT	15	1.49	1.56×10^{-4}
CSA	ACSA	18	1.47	5.67×10^{-4}
	CCSA	29	1.47	6.44×10^{-4}
CSS	SSJ	20	1.48	5.33×10^{-4}
	SFJ	40	1.49	5.91×10^{-4}
	ZJNFJ	35	1.48	6.25×10^{-4}
	HHA	19	1.48	7.38×10^{-4}

Supplementary Table 16. LD decay in each of the geographic groups

Group	SNP Group		
	r^2	Half distance (bp)	Number of Pairs
CT	0.342	5,600	24,114
ACSA	0.214	700	3,223,295
CCSA	0.202	1,200	4,982,059
HHA	0.184	1,600	3,304,603
SFJ	0.172	700	6,419,076
SSJ	0.190	3,300	4,204,654
ZJNFJ	0.174	800	6039,878

Supplementary Table 17. Fixation index (Fst) among different groups of tea populations

Population	CT	ACSA	CCSA	SSJ	SFJ	ZJNFJ	HHA
CT	0						
ACSA	0.244477	0					
CCSA	0.226413	0.087722	0				
SSJ	0.239998	0.152777	0.131797	0			
SFJ	0.233661	0.159139	0.149505	0.051333	0		
ZJNFJ	0.234969	0.162701	0.148436	0.028807	0.026164	0	
HHA	0.258015	0.164747	0.164747	0.028219	0.058015	0.028219	0

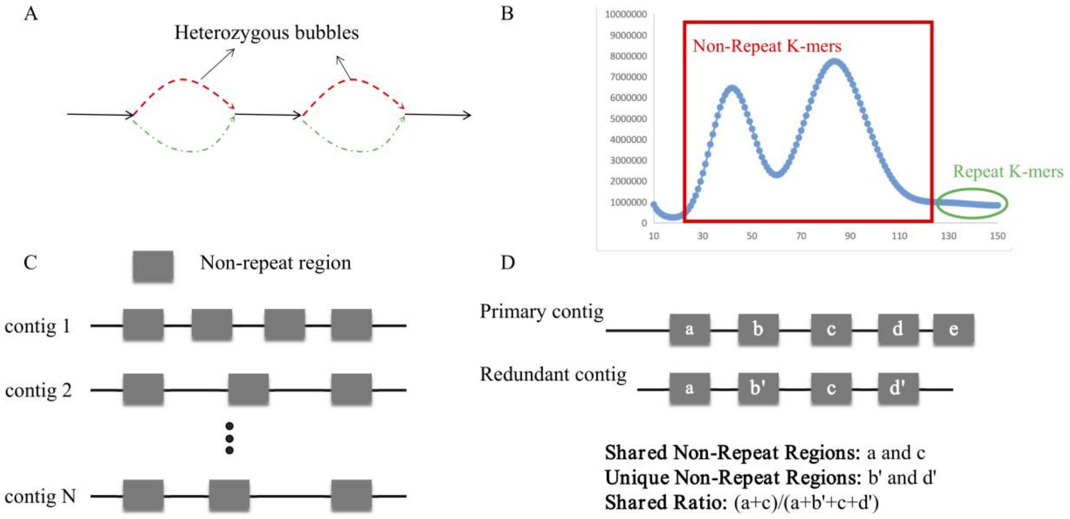

Supplementary Fig. 1. Illustration of Khaper algorithm for haplotype phasing. (A) Assembly of a highly heterozygous genome usually leads to bubbles, which represent phased contigs, due to high level of sequence variations. The black solid-line arrows represent haplotype-fused sequences, and the red and green dash-line arrows are haplotype resolved assembly. (B) K-mer distribution of a highly heterozygous diploid genome. Non-repeat K-mers highlighted in the red square and repeat K-mers in the green oval are identified using a cutoff of 1.5 × depth of the main peak. (C) Non-repeat regions are identified for each contig based on non-repeat K-mers. (D) Primary contigs and redundant sequences can be identified with pairwise comparison between contigs if they share a large proportion of Non-Repeat regions

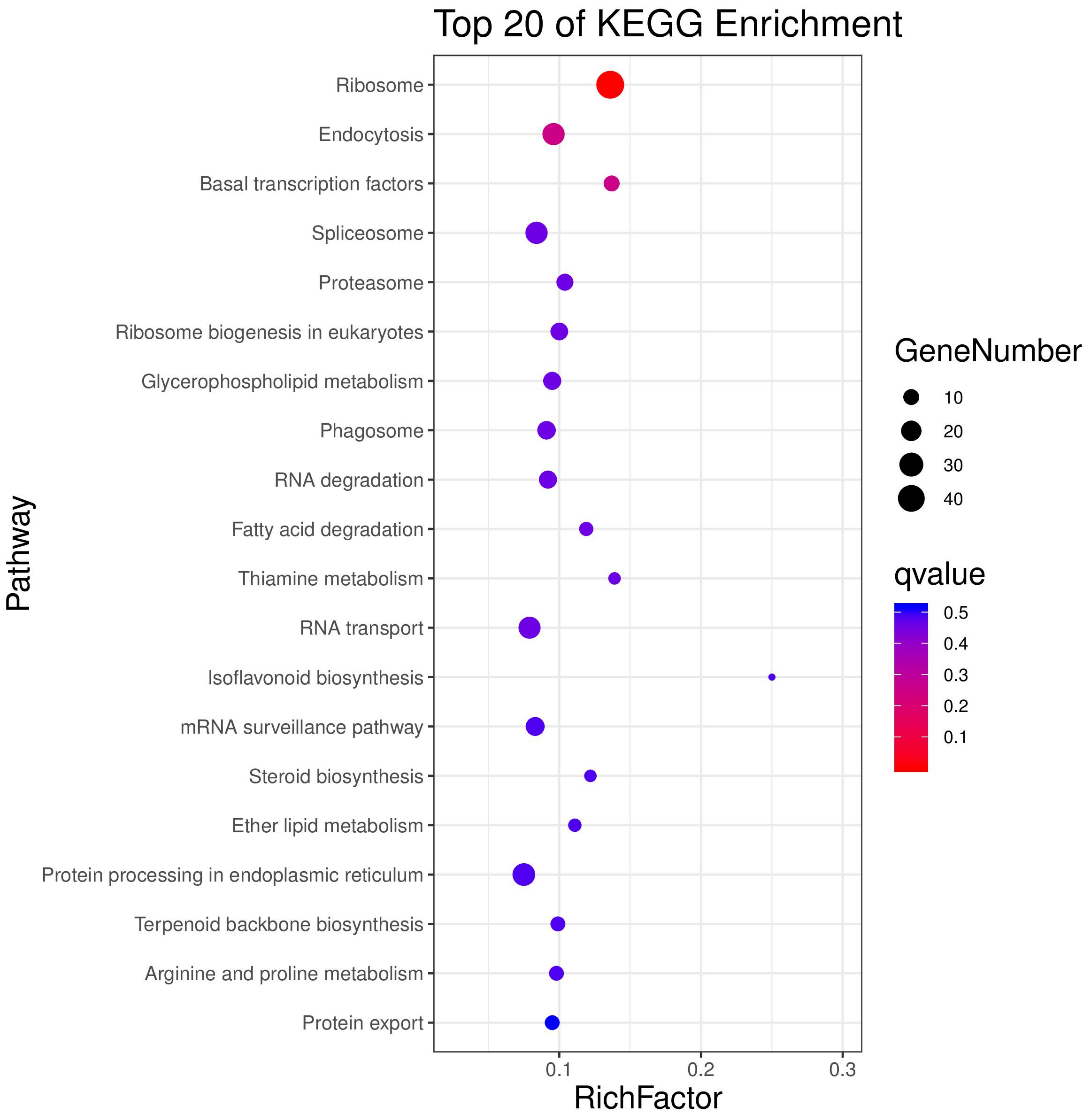

Supplementary Fig. 2. KEGG enrichment analysis of the genes with consistent ASE pattern.

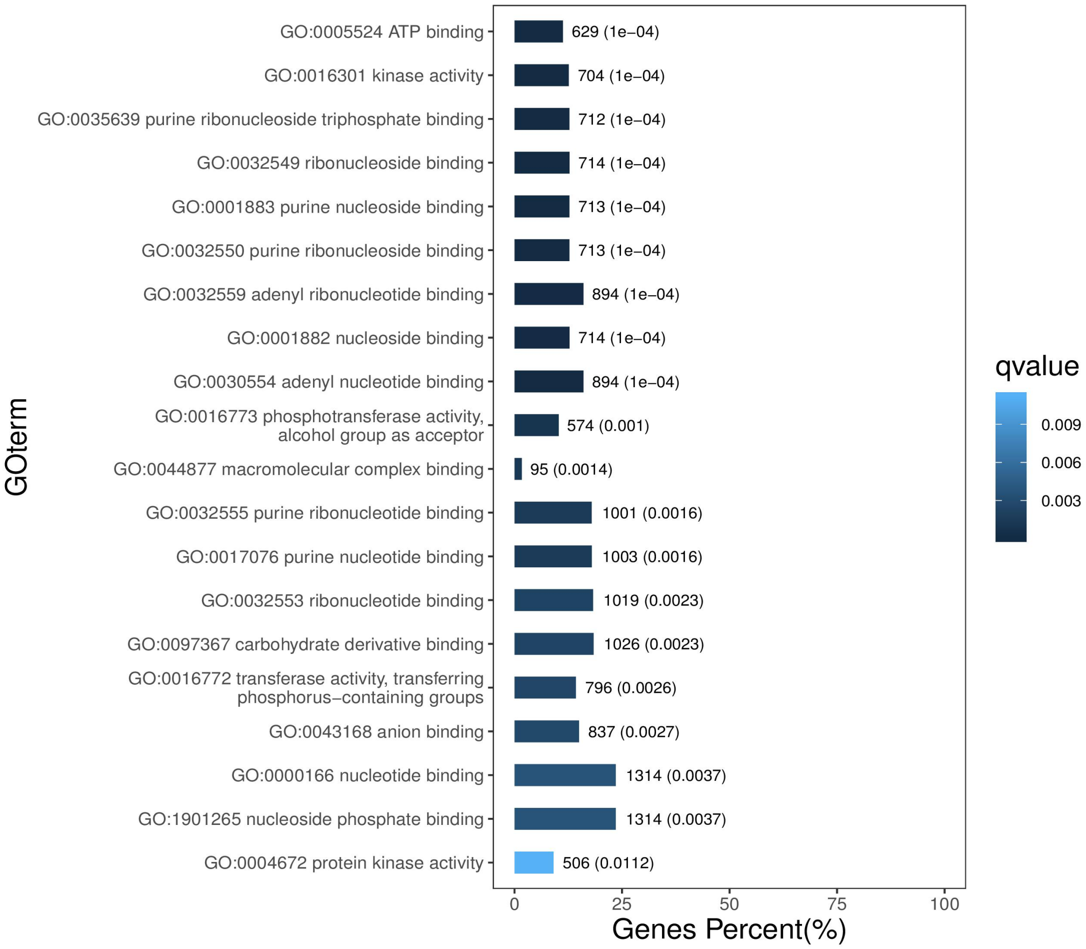

Supplementary Fig. 3. GO enrichment analysis of genes with large-effect variations

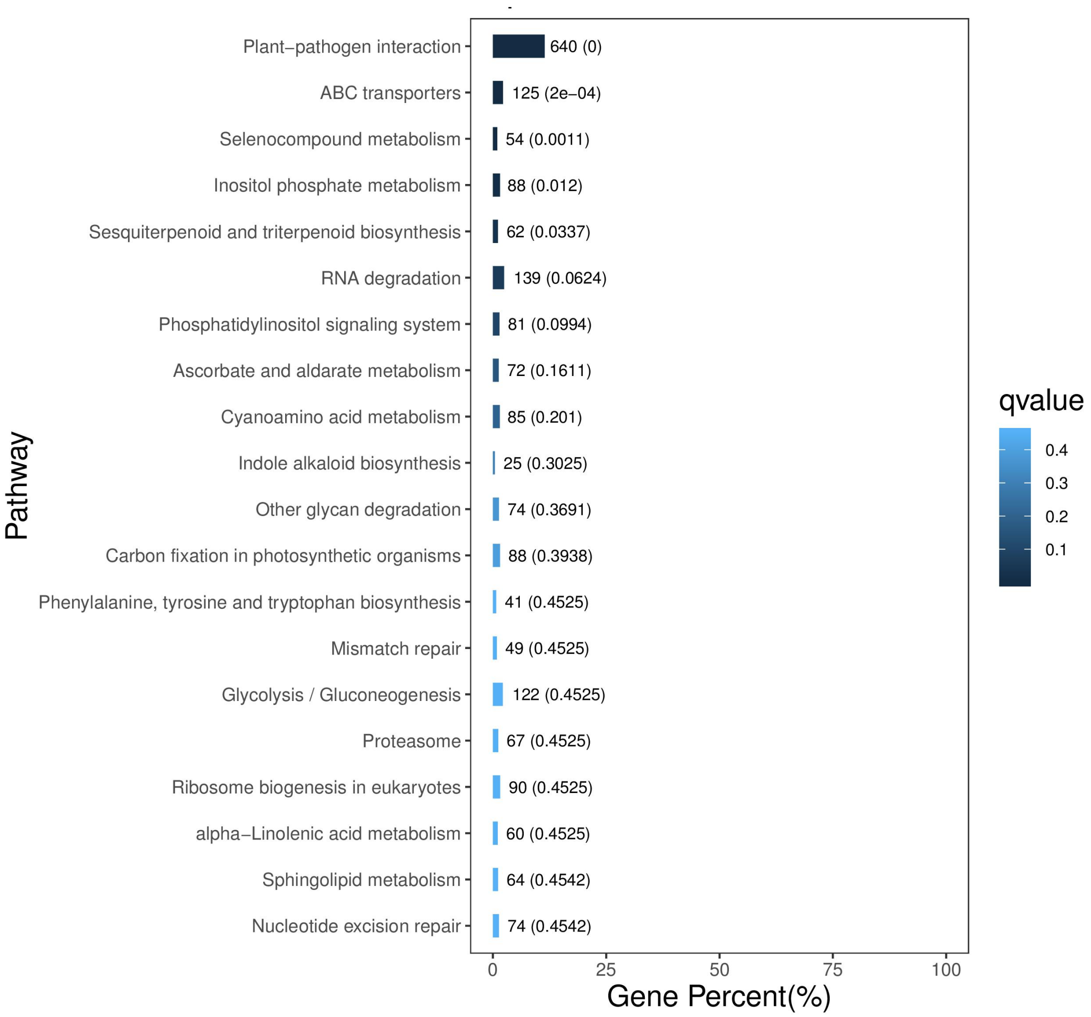

Supplementary Fig. 4. KEGG pathway enrichment analysis of genes with large-effect variation

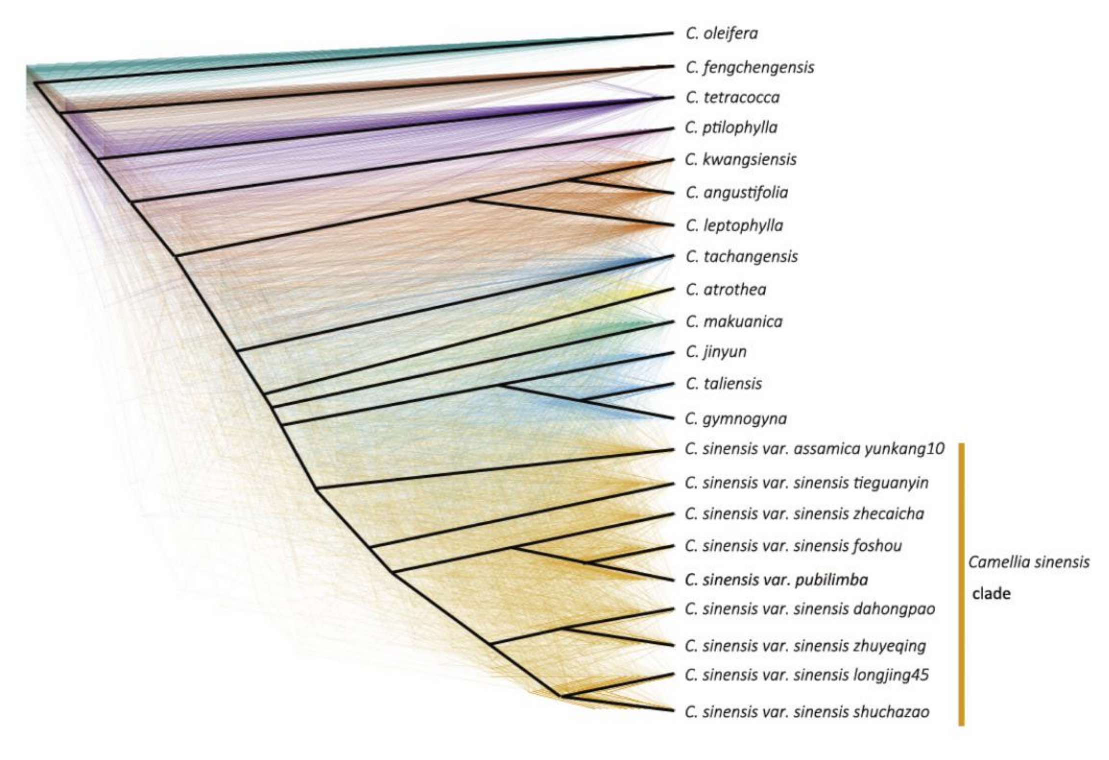

Supplementary Fig. 5. Densitree showing the discordance between 500 sampled gene trees and the species tree constructed using ASTRAL-III.

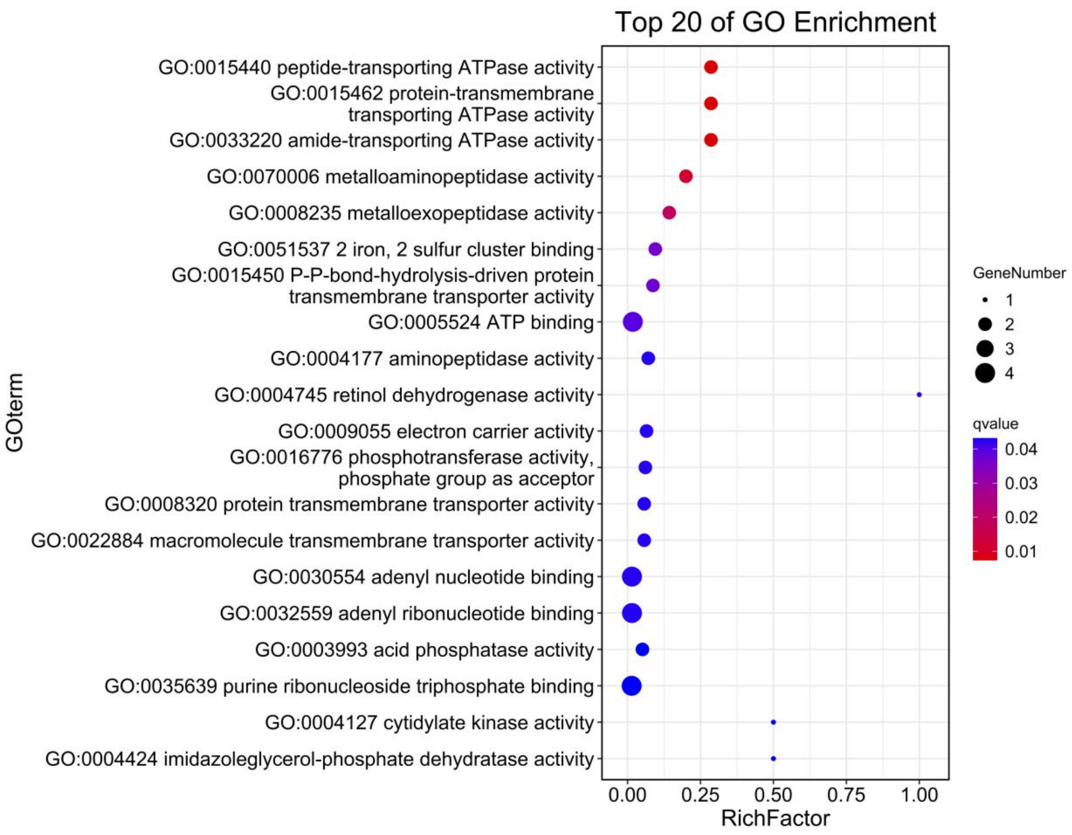

Supplementary Fig. 6. GO enrichment analysis of 98 introgressed genes that are shared in the six cultivated tea populations.

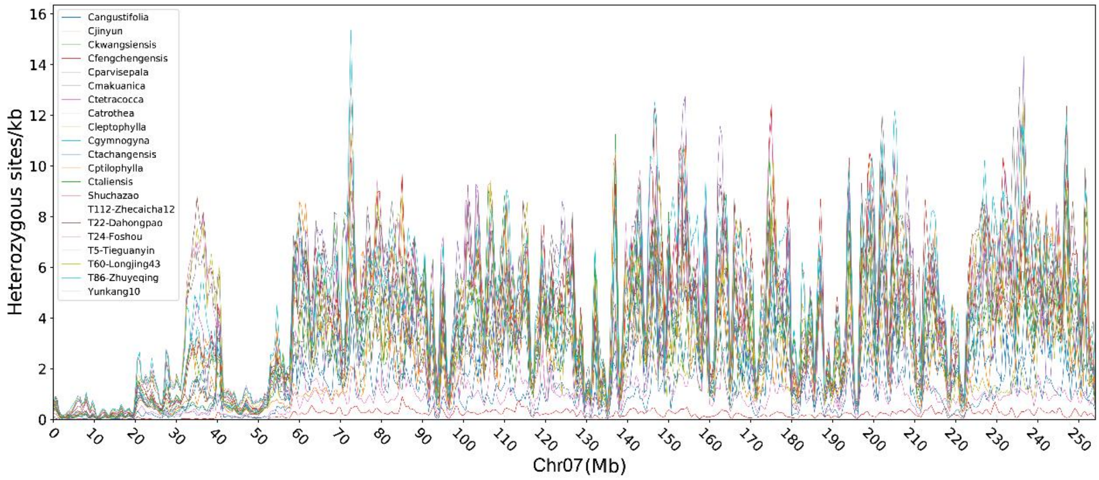

Supplementary Fig. 7. Distribution of heterozygous sites (per kb) of 21 resequenced individuals along chromosome 07, including 12 close relatives, seven var. sinensis and one var. assamica.

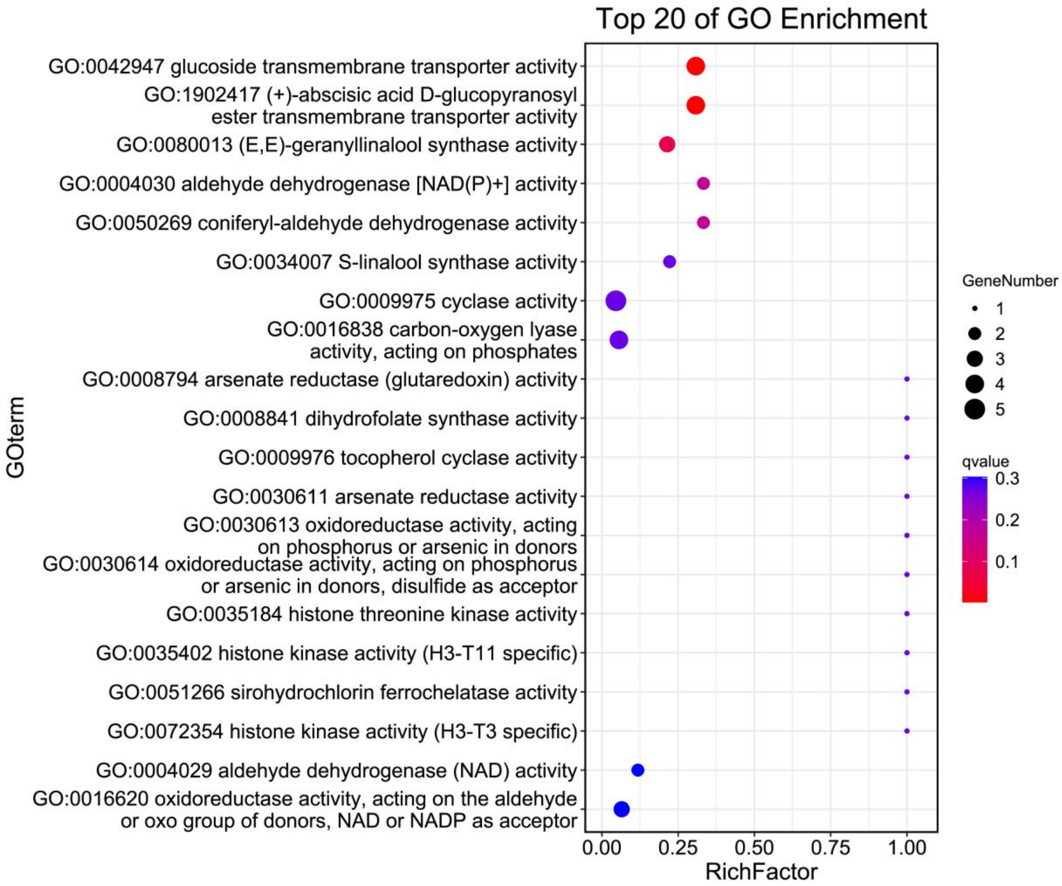

Supplementary Fig. 8. GO enrichment of artificially selected protein-coding genes in the early domestication process of CSA landraces.

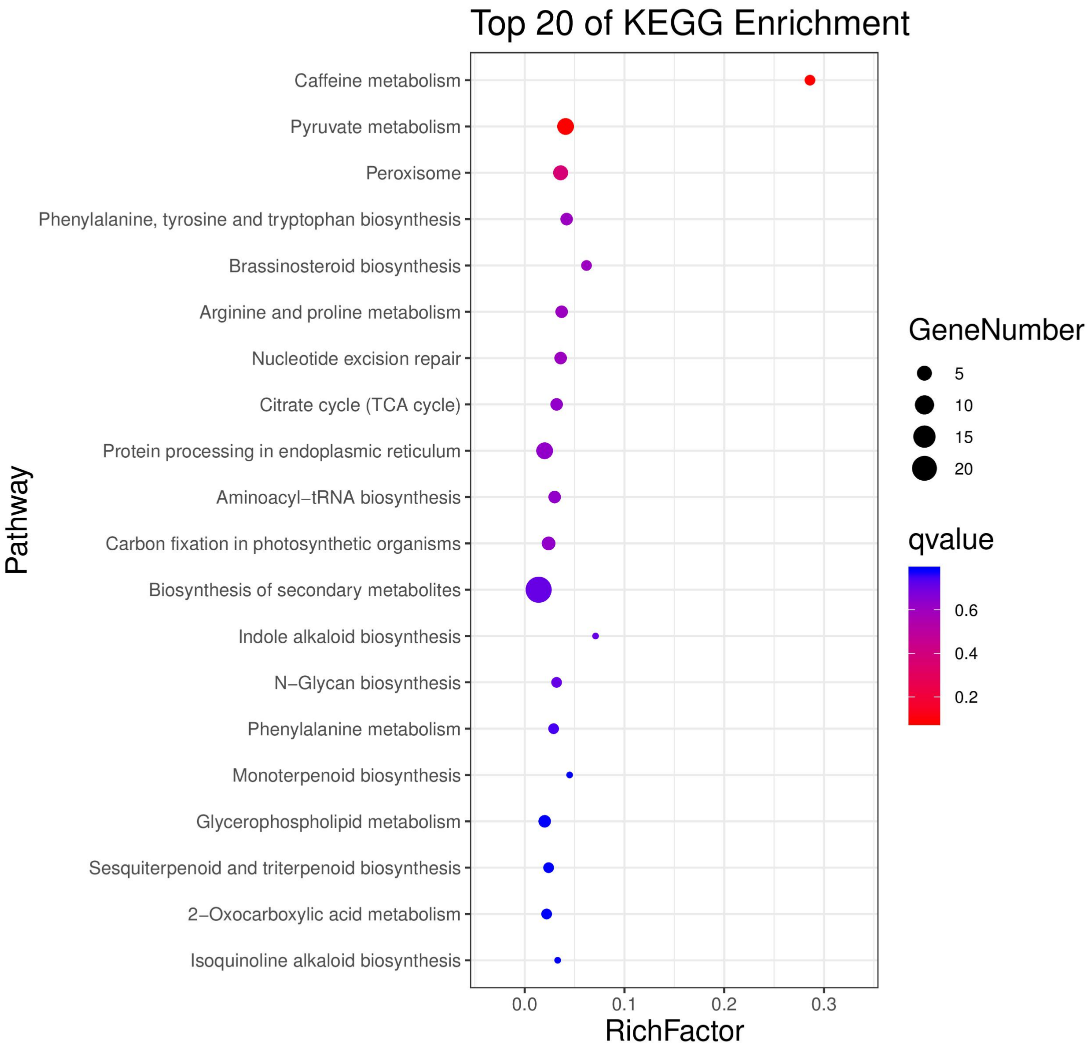

Supplementary Fig. 9. Top 20 pathways based on the KEGG enrichment analysis of artificially selected genes in the improvement of CSA elite cultivars.

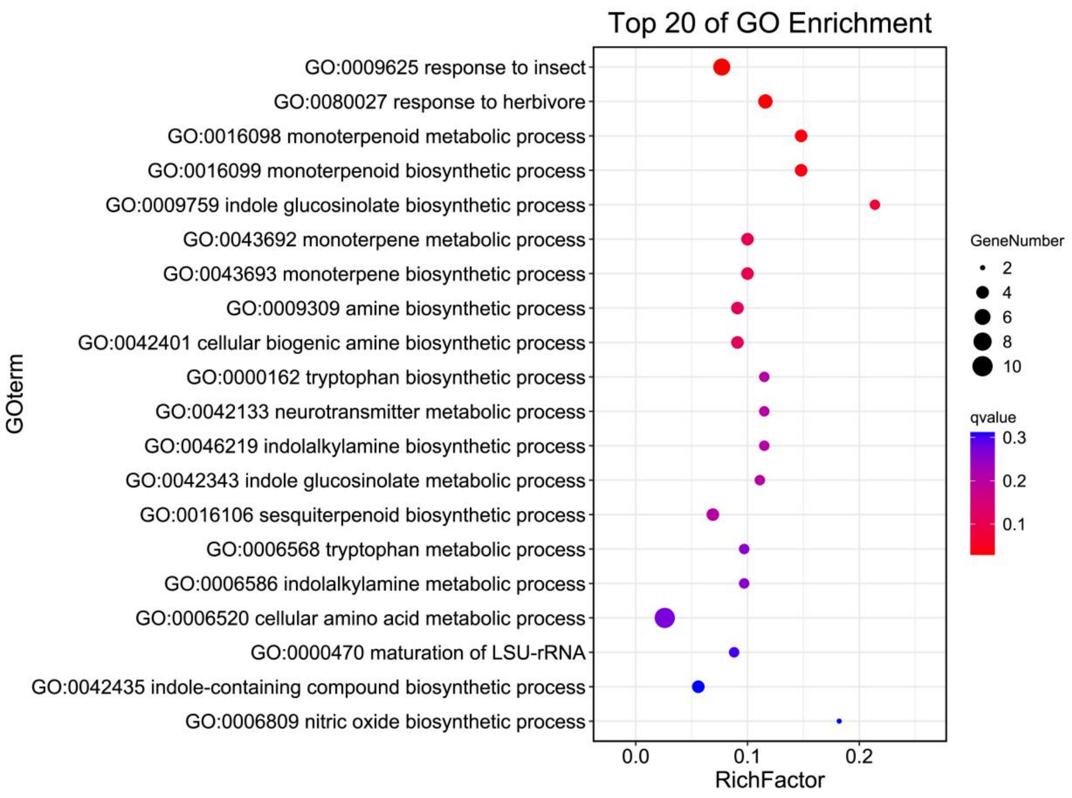

Supplementary Fig. 10. GO enrichment (Biological Process) of artificially selected protein-coding genes in the early domestication process of CSS landraces .

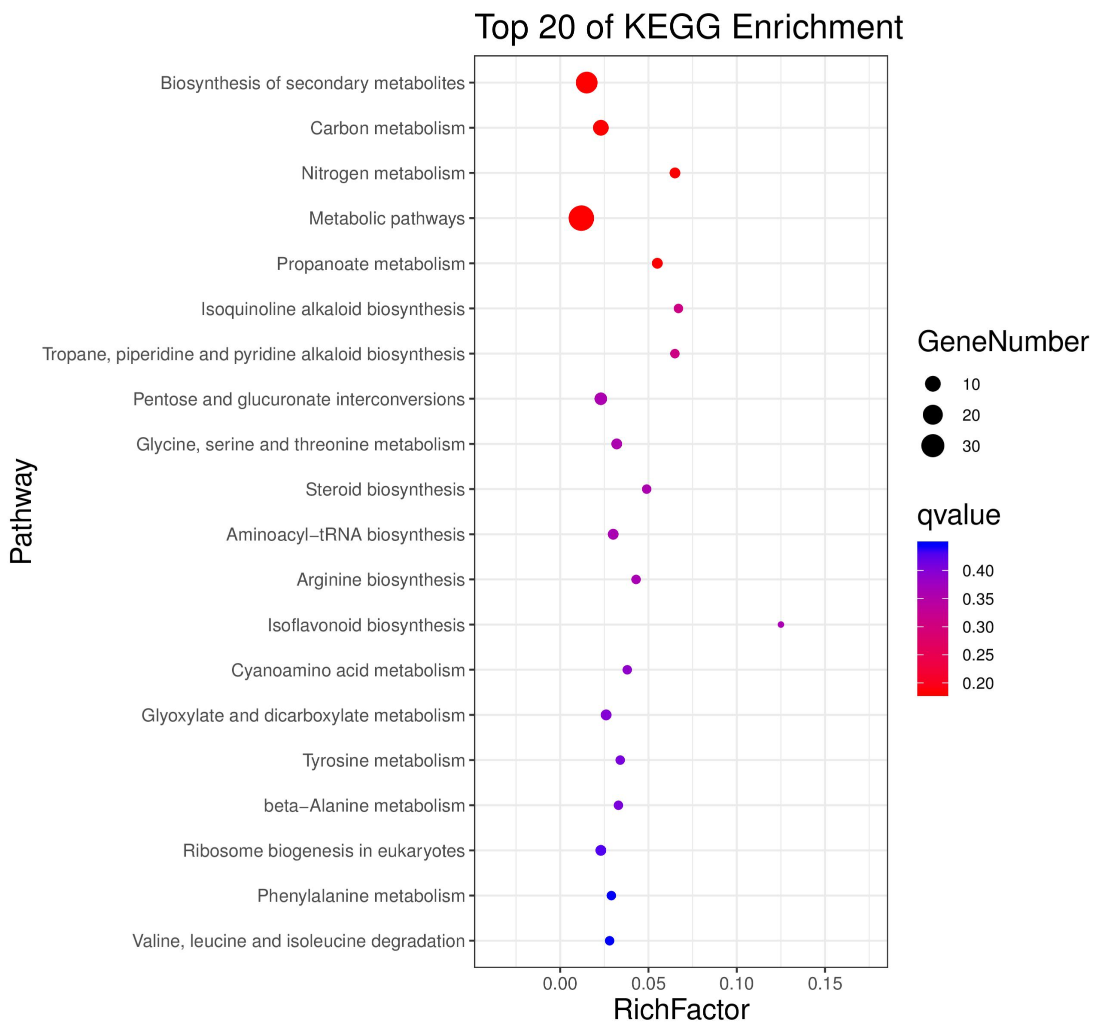

Supplementary Fig. 11. Top 20 pathways based on the KEGG enrichment analysis of artificially selected genes in the early domestication of CSS landraces.

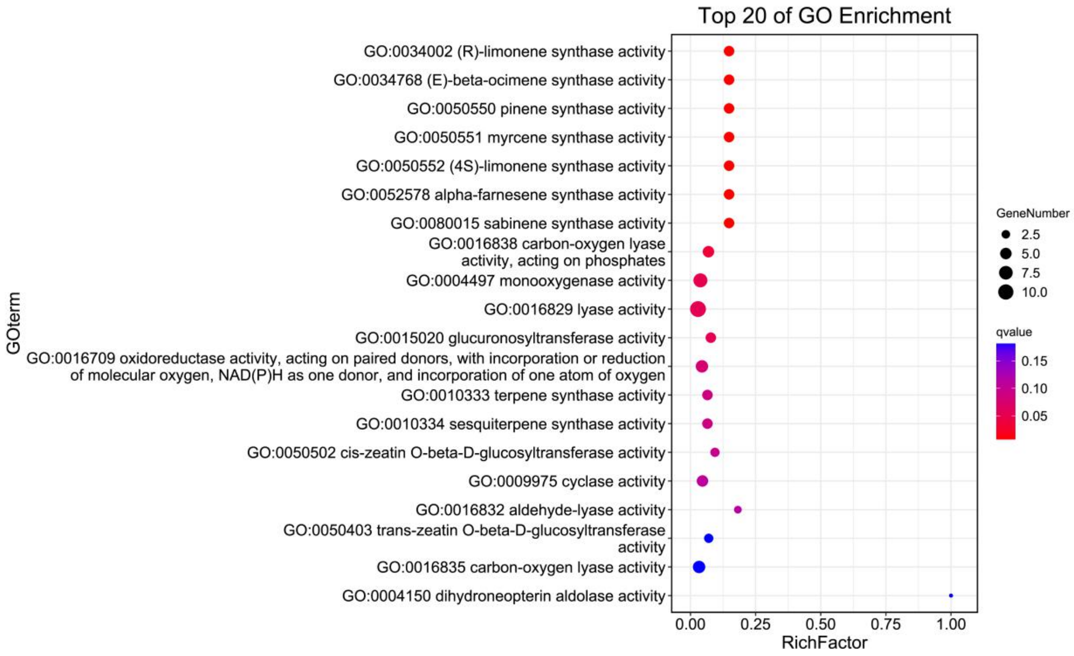

Supplementary Fig. 12. GO enrichment (Molecular Function) of artificially selected protein-coding genes in the early domestication process of CSS landraces .

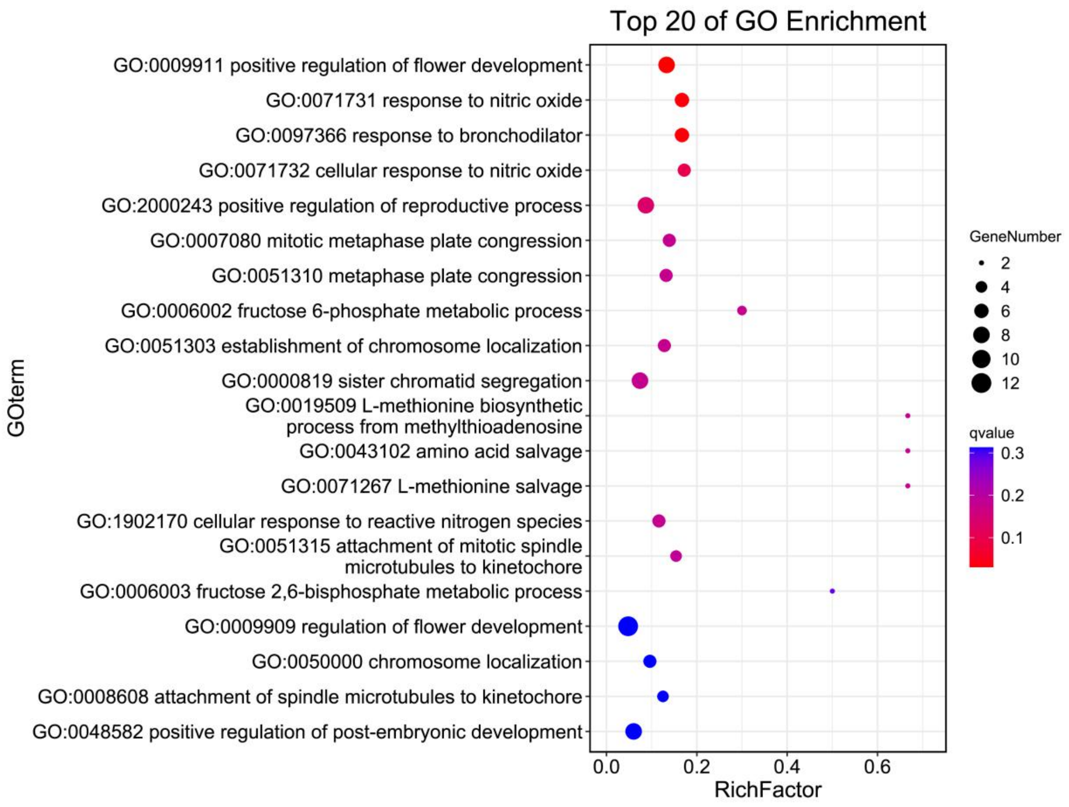

Supplementary Fig. 13. GO enrichment (Biological Process) of artificially selected protein-coding genes in the improvement process of CSS elite.
