# Supplementary material for: Haplotype-resolved genome assembly provides insights into evolutionary history of the tea plant Camellia sinensis
Source: Nat Genet. 2021 Jul 15;53(8):1250–9. doi: 10.1038/s41588-021-00895-y (PMC8346365; doi:10.1038/s41588-021-00895-y)
Supplement: Supplementary file 2 — Reporting Summary [file 41588_2021_895_MOESM2_ESM.pdf]

nature research

Corresponding author(s): Xingtan Zhang

Last updated by author(s): May 31, 2021

Reporting Summary

Nature Research wishes to improve the reproducibility of the work that we publish. This form provides structure for consistency and transparency in reporting. For further information on Nature Research policies, see Authors & Referees and the Editorial Policy Checklist.

Statistics

For all statistical analyses, confirm that the following items are present in the figure legend, table legend, main text, or Methods section.

n/a	Confirmed	
☐	☒	The exact sample size (*n*) for each experimental group/condition, given as a discrete number and unit of measurement
☐	☒	A statement on whether measurements were taken from distinct samples or whether the same sample was measured repeatedly
☐	☒	The statistical test(s) used AND whether they are one- or two-sided<br>*Only common tests should be described solely by name; describe more complex techniques in the Methods section.*
☒	☐	A description of all covariates tested
☐	☒	A description of any assumptions or corrections, such as tests of normality and adjustment for multiple comparisons
☒	☐	A full description of the statistical parameters including central tendency (e.g. means) or other basic estimates (e.g. regression coefficient) AND variation (e.g. standard deviation) or associated estimates of uncertainty (e.g. confidence intervals)
☐	☒	For null hypothesis testing, the test statistic (e.g. *F*, *t*, *r*) with confidence intervals, effect sizes, degrees of freedom and *P* value noted<br>*Give P values as exact values whenever suitable.*
☒	☐	For Bayesian analysis, information on the choice of priors and Markov chain Monte Carlo settings
☒	☐	For hierarchical and complex designs, identification of the appropriate level for tests and full reporting of outcomes
☒	☐	Estimates of effect sizes (e.g. Cohen's *d*, Pearson's *r*), indicating how they were calculated

Our web collection on statistics for biologists contains articles on many of the points above.

Software and code

Policy information about availability of computer code

Data collection	The sequencing reads were undertaken with PacBio Sequel 2 and Illumina NovaSeq platforms.
Data analysis	Khaper algorithm is freely available in GitHub (https://github.com/lardo/khaper) and calc_switchErr can be found in GitHub (https://github.com/tangerzhang/calc_switchErr/). The codes (Khaper and calc_switchErr) also have been archived to Zenodo with the DOIs (10.5281/zenodo.4780792 and 10.5281/zenodo.4780666) and cited in refs.15 and 19.<br>Other programs used in this study are listed as follows:<br>CANU (version 1.9), BWA (version 0.7.15-r1140), Pilon (version 1.22), ALLHiC (version 0.1 https://github.com/tangerzhang/ALLHiC), Purge_haplotigs (https://bitbucket.org/mroachawri/purge_haplotigs/src/master/), Pseudohaploid (https://github.com/schatzlab/pseudohaploid), 3D-DNA (version 180922), GATK (version 3.8), SAMtools (version 1.9), VCFtools (version 0.1.16), BLASTN (version 2.7.1), BLASTP (version 2.7.1), LAST (version 959), R (version 3.5.1), BUSCO (version 3.0.2), LTR_retriever (version 2.8), RECON (version 1.08), RepeatScout (version 1.0.5), TEclass (version 2.1.3), TRF (version 4.07), LTR-FINDER (version 1.0.5), RepeatMasker (version open-4.0.7), LTRharvest (version 1.5.10), GeneWise (version 2.4.1), Trinity (version 2.3.2), RSEM (version 1.2.31), PASA (version 2.2.0), Augustus (version 2.4), SNAP(https://github.com/KorfLab/SNAP), BLAT (version 350), MAKER (version 2.31.10), OrthoMCL (version 1.1.4), MUSCLE (version 3.8.31), DensiTree (version 2.2.5),, MCScanX (http://chibba.pgml.uga.edu/mcscan2/), MCscan (https://github.com/tanghaibao/jcvi/wiki/MCscan-(Python-version)), iTOL (https://itol.embl.de/), proc10xG (https://github.com/ucdavis-bioinformatics/proc10xG), NUCMER (version. 4.0.0), GMAP (version 2013-10-28), MAFFT (version 7.299b), Trimmomatic (version 0.33), IQ-Tree (version 1.6.12), RAxML (version 8.2.12), PLINK (version 1.9), ANGSD (version0.932), PopLDdecay (version 3.31), ADMIXTURE (version 1.3.0), Selscan (version 1.3.0).

For manuscripts utilizing custom algorithms or software that are central to the research but not yet described in published literature, software must be made available to editors/reviewers. We strongly encourage code deposition in a community repository (e.g. GitHub). See the Nature Research guidelines for submitting code & software for further information.

Data

Policy information about availability of data

All manuscripts must include a data availability statement. This statement should provide the following information, where applicable:

- Accession codes, unique identifiers, or web links for publicly available datasets
- A list of figures that have associated raw data
- A description of any restrictions on data availability

The raw sequencing reads of PacBio, Illumina, 10× Genomics, Hi-C, RNA-seq and ISO-seq have been deposited in the National Center for Biotechnology Information (NCBI) database with the accession number PRJNA665594 and/or in GSA database (https://bigd.big.ac.cn/gsa/) under the accession number PRJCA003090. The assembly and annotation have been archived in NCBI with the accession number JAFLEL000000000 and GWH (https://bigd.big.ac.cn/gwh/) with the accession numbers GWHASIV00000000 for the monoploid and GWHASIX00000000 for the haplotype-resolved genomes. The VCF that contains all of the clean SNPs were uploaded to Mendeley database (https://data.mendeley.com/datasets/7hb33vd7sf/1). In addition, three datasets that were used to assess the switch errors in the haplotype-resolved TGY genome assembly were deposited to Mendeley database (http://dx.doi.org/10.17632/xpccyg5w2x.1).

Field-specific reporting

Please select the one below that is the best fit for your research. If you are not sure, read the appropriate sections before making your selection.

☒ Life sciences ☐ Behavioural & social sciences ☐ Ecological, evolutionary & environmental sciences

For a reference copy of the document with all sections, see nature.com/documents/nr-reporting-summary-flat.pdf

Life sciences study design

All studies must disclose on these points even when the disclosure is negative.

Sample size	Samples were selected to have enough representation from cultivated and wild accessions to query meaningful and decisive conclusion. The germplasm collected in our study cover majority of the growing regions across China.
Data exclusions	No data were excluded.
Replication	Expression were repeated three times and all attempts at replication were successful.
Randomization	This is not relevant to our study as all of the analysis in the research followed published methods rather than random selection.
Blinding	The investigators were blinded to group allocation during the data collection

Reporting for specific materials, systems and methods

We require information from authors about some types of materials, experimental systems and methods used in many studies. Here, indicate whether each material, system or method listed is relevant to your study. If you are not sure if a list item applies to your research, read the appropriate section before selecting a response.

Materials & experimental systems

n/a	Involved in the study	
☒	☐	Antibodies
☒	☐	Eukaryotic cell lines
☒	☐	Palaeontology
☒	☐	Animals and other organisms
☒	☐	Human research participants
☒	☐	Clinical data

Methods

n/a	Involved in the study	
☒	☐	ChIP-seq
☒	☐	Flow cytometry
☒	☐	MRI-based neuroimaging
